# GTPase Activity and Neuronal Toxicity of Parkinson's Disease–Associated LRRK2 Is Regulated by ArfGAP1

**DOI:** 10.1371/journal.pgen.1002526

**Published:** 2012-02-09

**Authors:** Klodjan Stafa, Alzbeta Trancikova, Philip J. Webber, Liliane Glauser, Andrew B. West, Darren J. Moore

**Affiliations:** 1 Laboratory of Molecular Neurodegenerative Research, Brain Mind Institute, School of Life Sciences, Ecole Polytechnique Fédérale de Lausanne (EPFL), Lausanne, Switzerland; 2 Department of Neurology, Center for Neurodegeneration and Experimental Therapeutics, University of Alabama at Birmingham, Birmingham, Alabama, United States of America; University of Minnesota, United States of America

## Abstract

Mutations in the *leucine-rich repeat kinase 2* (*LRRK2*) gene are the most common cause of autosomal dominant familial Parkinson's disease (PD) and also contribute to idiopathic PD. *LRRK2* encodes a large multi-domain protein with GTPase and kinase activity. Initial data indicates that an intact functional GTPase domain is critically required for LRRK2 kinase activity. PD–associated mutations in LRRK2, including the most common G2019S variant, have variable effects on enzymatic activity but commonly alter neuronal process morphology. The mechanisms underlying the intrinsic and extrinsic regulation of LRRK2 GTPase and kinase activity, and the pathogenic effects of familial mutations, are incompletely understood. Here, we identify a novel functional interaction between LRRK2 and ADP-ribosylation factor GTPase-activating protein 1 (ArfGAP1). LRRK2 and ArfGAP1 interact *in vitro* in mammalian cells and *in vivo* in brain, and co-localize in the cytoplasm and at Golgi membranes. PD–associated and functional mutations that alter the GTPase activity of LRRK2 modulate the interaction with ArfGAP1. The GTP hydrolysis activity of LRRK2 is markedly enhanced by ArfGAP1 supporting a role for ArfGAP1 as a GTPase-activating protein for LRRK2. Unexpectedly, ArfGAP1 promotes the kinase activity of LRRK2 suggesting a potential role for GTP hydrolysis in kinase activation. Furthermore, LRRK2 robustly and directly phosphorylates ArfGAP1 *in vitro*. Silencing of ArfGAP1 expression in primary cortical neurons rescues the neurite shortening phenotype induced by G2019S LRRK2 overexpression, whereas the co-expression of ArfGAP1 and LRRK2 synergistically promotes neurite shortening in a manner dependent upon LRRK2 GTPase activity. Neurite shortening induced by ArfGAP1 overexpression is also attenuated by silencing of LRRK2. Our data reveal a novel role for ArfGAP1 in regulating the GTPase activity and neuronal toxicity of LRRK2; reciprocally, LRRK2 phosphorylates ArfGAP1 and is required for ArfGAP1 neuronal toxicity. ArfGAP1 may represent a promising target for interfering with LRRK2-dependent neurodegeneration in familial and sporadic PD.

## Author Summary

Parkinson's disease (PD) is the most common neurodegenerative movement disorder. Current therapies for treating PD are symptomatic and rely on restoring dopamine signaling. There is presently no cure for PD. PD is typically a sporadic disease, although 5%–10% of cases are known to have a familial origin. Mutations in at least seven genes are known to cause familial forms of PD, with mutations in the *leucine-rich repeat kinase 2* (*LRRK2*) gene at the *PARK8* locus representing the most common cause of familial and sporadic PD. The *LRRK2* gene encodes a multi-domain protein with two enzymatic activities, GTPase and kinase, and familial mutations are known to variably influence these activities. Familial mutations in LRRK2 promote toxicity in cultured neurons, which is dependent on both GTPase and kinase activity. The factors regulating the GTPase activity of LRRK2 are poorly understood. Here, we identify the ArfGAP1 protein as a novel regulator of LRRK2 GTPase and kinase activity as well as neuronal toxicity induced by LRRK2. ArfGAP1 also serves as a novel substrate for phosphorylation mediated by LRRK2 kinase activity. ArfGAP1 may therefore represent a promising molecular target for interfering with neurodegeneration due to *LRRK2* mutations in familial and sporadic forms of PD.

## Introduction

Mutations in the *leucine-rich repeat kinase 2* gene (LRRK2, PARK8, OMIM 607060) cause late-onset, autosomal dominant Parkinson's disease (PD) that is clinically and neurochemically indistinguishable from idiopathic PD [1], [2], [3]. *LRRK2* mutations that clearly segregate with disease include R1441C/G/H, Y1699C, G2019S and I2020T [3]. Importantly, the G2019S mutation has also been identified in subjects with idiopathic PD at varying frequencies depending on ethnicity thereby linking LRRK2 dysfunction to idiopathic disease [4], [5], [6]. The *LRRK2* gene encodes a large protein of 2527 amino acids belonging to the ROCO protein family [7]. Similar to other ROCO proteins, LRRK2 contains a Ras-of-complex (Roc) GTPase domain and a C-terminal of Roc (COR) domain in conjunction with a protein kinase domain with closest homology to members of the mixed-lineage and receptor-interacting protein kinase families. LRRK2 also contains a number of repeat domains of undetermined function including N-terminal LRRK2-specific, armadillo, ankyrin and leucine-rich repeats and C-terminal WD40 repeats that surround the central Roc-COR-kinase catalytic region. LRRK2 can function *in vitro* as a kinase whereby it can mediate autophosphorylation and can phosphorylate generic substrates (i.e. myelin basic protein or LRRKtide) or putative substrates such as 4E-BP1, moesin, β-tubulin and FoxO1 [8], [9], [10], [11], [12], [13], [14]. It is not yet clear whether LRRK2 acts as a kinase *in vivo* to phosphorylate physiological or pathological substrates [15]. The GTPase domain of LRRK2 can bind to GDP or GTP via a guanine nucleotide phosphate-binding loop (P-loop), and can hydrolyze GTP, albeit at a relatively slow rate, via an adjacent Switch II catalytic region [16], [17], [18], [19], [20]. It has been proposed that GTP binding at the GTPase domain may enhance the kinase activity of LRRK2 whereas numerous studies have shown that mutations (i.e. K1347A or T1348N) which prevent the binding of guanine nucleotides to the P-loop region abolish kinase activity [19], [20]. A recent study has demonstrated that LRRK2 kinase activity is independent of GTP binding *per se* but is instead dependent on the capacity for GTP binding thus potentially suggesting the GTP-dependent activation of LRRK2 by an upstream guanine nucleotide-binding protein [21]. These studies potentially support a model whereby LRRK2 is a GTPase-regulated protein kinase although whether kinase activity serves as the main output of LRRK2 is not clear. Autophosphorylation of residues within the GTPase domain of LRRK2 could suggest a reciprocal regulation of GTPase activity by kinase activity [22], [23], [24], [25]. At present, it is unclear how the GTPase activity of LRRK2 is regulated. Similar to other small GTPases, it is assumed that guanine nucleotide exchange factors (GEFs) promote the exchange of GDP for GTP within the LRRK2 GTPase domain whereas GTPase-activating proteins (GAPs) promote the hydrolysis of LRRK2-bound GTP. A putative GEF for LRRK2, ARHGEF7, has recently been identified although as yet no GAPs have been nominated [26]. The absence of these factors may explain why LRRK2 exhibits such poor GTPase activity *in vitro* [16], which has hampered the characterization of this important functional domain.

Disease-associated mutations in LRRK2 located throughout the central Roc-COR-kinase region have variable effects on GTPase and kinase activity. The R1441C, R1441G and Y1699C variants lead to a modest impairment of GTP hydrolysis and produce a resulting increase in steady-state GTP binding [17], [18], [27], [28], but without consistent effects on kinase activity [9], [10], [12], [20]. In contrast, the common G2019S variant markedly enhances kinase activity but does not appear to regulate GTPase activity [9], [10], [12], [20], [27]. Therefore, the precise actions of familial mutations on LRRK2 enzymatic activity are incompletely understood although it is clear that alterations in both GTPase and kinase activities are important for the development of PD. Despite the differential effects on LRRK2 activity, familial mutations commonly promote LRRK2-induced cellular toxicity. Studies in cultured primary cortical neurons, and some neural cell lines, reveal that the exogenous expression of full-length human LRRK2 harboring familial mutations (i.e. R1441C, Y1699C and G2019S) induces marked neuronal toxicity and cell death relative to the wild-type protein [9], [19], [20], [29], [30], [31]. Preventing GDP/GTP binding or impairing kinase activity attenuates LRRK2-induced neuronal toxicity, at least for the R1441C and G2019S variants [19]. *In vivo*, viral-mediated gene transfer models reveal that expression of G2019S but not wild-type LRRK2 induces the degeneration of nigrostriatal dopaminergic neurons in rodents which can be prevented by pharmacological or genetic inhibition of kinase activity [32], [33]. A role for GTPase activity or other familial mutations in LRRK2-induced toxicity in viral rodent models *in vivo* has not yet been evaluated, although the expression of R1441G or R1441C variants in transgenic mice can produce neuropathological phenotypes [34], [35]. Certain familial mutations (i.e. G2019S and I2020T) have also been shown to enhance LRRK2-induced shortening of neuronal processes in cultured neurons compared to wild-type LRRK2, whereas ablation of endogenous LRRK2 expression oppositely enhances neurite length [36], [37], [38]. The effects of the G2019S variant on neurite morphology are dependent on kinase activity and are mediated by autophagy whereas the contribution of LRRK2 GTPase activity is not known [36], [37]. Therefore, the exact contribution of GTPase activity to LRRK2-dependent neuronal phenotypes is poorly characterized at present. Given that GTPase activity is critically required for regulating the kinase activity and neurotoxicity of LRRK2 [19], [20], it is important to clarify the intrinsic and extrinsic factors that regulate GTPase activity.

We recently developed a simple model of LRRK2-induced toxicity in the baker's yeast, *Saccharomyces cerevisiae* [27]. LRRK2 toxicity in this yeast model, as well as in primary cortical neurons, is regulated by GTPase activity and correlates with impairments in the trafficking of endosomes and autophagic vacuoles. A genome-wide genetic screen in this model identified nine yeast genes that regulate LRRK2-induced toxicity and trafficking defects. Here, we evaluate the mammalian ortholog of one of these genetic modifiers in regulating the function of LRRK2. We identify ArfGAP1, an ortholog of yeast *GCS1*, as a novel GAP for regulating LRRK2 GTPase activity and neuronal toxicity.

## Results

### Interaction of LRRK2 with ArfGAP1 in cells and *in vivo*

A recent genetic modifier screen in yeast identified nine genes that when deleted can modulate the toxicity induced by expression of human LRRK2 [27]. Seven mutants suppressed and two mutants enhanced LRRK2 toxicity. We sought to determine whether these genes are relevant for the effects of LRRK2 in the context of mammalian cells. However, of the nine modifiers, only two yeast genes (*SLT2* and *GCS1*) which acted to suppress toxicity when deleted possess mammalian orthologs. Yeast *SLT2* encodes a serine/threonine mitogen-activated protein kinase (MAPK) with broad similarity to ERK, p38 and JNK MAPKs. We have previously shown that LRRK2 expression exhibits modest effects on MAPK signaling in mammalian cells in a kinase-independent manner [20]. Yeast *GCS1* encodes an ADP-ribosylation factor (Arf) GTPase-activating protein (GAP) with a single mammalian ortholog, ArfGAP1. Therefore, we decided to focus on exploring the functional relationship between LRRK2 and ArfGAP1. *ArfGAP1* encodes a 415 amino-acid protein that functions as a GAP to promote the GTP hydrolysis of Arf1, a small GTPase that is critical for maintaining normal Golgi morphology [39], [40], [41], [42]. Arf1 GTP hydrolysis is required for the dissociation of coat proteins from Golgi-derived membranes and vesicles [43]. ArfGAP1 contains an N-terminal GAP domain (∼130 amino acids) comprising a C4-type zinc finger motif (residues 22–45) which promotes the hydrolysis of Arf1-bound GTP, and a C-terminal non-catalytic domain involved in Golgi membrane localization and protein-protein interactions namely with the KDEL receptor [43].

**Figure 1:**
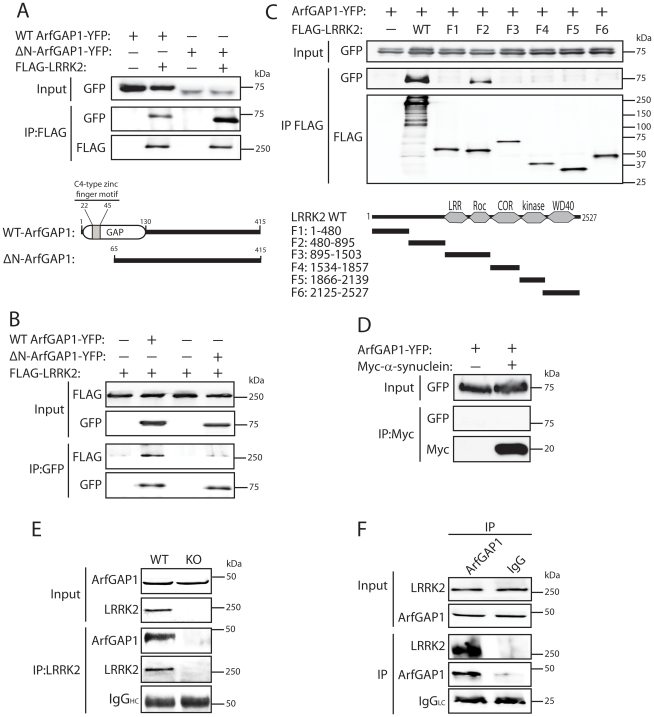
LRRK2 interacts with ArfGAP1 in mammalian cells and *in vivo*. (A) YFP-tagged ArfGAP1 (WT or ΔN) interacts with full-length FLAG-tagged LRRK2 following immunoprecipitation (IP) with anti-FLAG antibody from HEK-293T cells. Domain organization of ArfGAP1 constructs is indicated. (B) Interaction of ArfGAP1-YFP (WT or ΔN) and FLAG-LRRK2 following reverse IP with anti-GFP antibody from HEK-293T cells. (C) Domain mapping reveals the interaction of ArfGAP1-YFP with full-length (WT) LRRK2 and residues 480–895 of LRRK2 (fragment F2) following IP with anti-FLAG antibody from HEK-293T cells. Domain organization of LRRK2 deletion mutants is indicated. (D) Myc-α-synuclein fails to interact with ArfGAP1-YFP following IP with anti-myc antibody from HEK-293T cells. (E) LRRK2 interacts with ArfGAP1 in cerebral cortex extracts from WT mice but not LRRK2 knockout (KO) mice following IP with anti-LRRK2 antibody (clone c41-2). (F) ArfGAP1 interacts with LRRK2 in mouse cerebral cortex following IP with anti-ArfGAP1 antibody but not with a rabbit IgG control. IgG light chain (LC) indicates the equivalent amounts of IgG used for IP. Molecular mass markers are indicated in kilodalton (kDa). Data are representative of at least two independent experiments.

To begin to explore the potential relationship between LRRK2 and ArfGAP1, the interaction of these two proteins was assessed by co-immunoprecipitation in HEK-293T cells expressing FLAG-tagged LRRK2 and YFP-tagged ArfGAP1. Following immunoprecipitation of full-length LRRK2 we find a robust interaction with full-length ArfGAP1 and with an N-terminal deletion mutant lacking amino acids 1–64 (ΔN-ArfGAP1) which exhibits negligible GAP activity (Figure 1A) [39]. In the reverse experiment, immunoprecipitation of full-length and ΔN-ArfGAP1 reveals an interaction with full-length LRRK2 (Figure 1B). Thus, LRRK2 most likely interacts with the C-terminal non-catalytic domain of ArfGAP1. To determine whether the interaction between LRRK2 and ArfGAP1 is direct, *in vitro* pull-down assays were conducted between immunopurified full-length FLAG-tagged LRRK2 or parkin and recombinant full-length GST-tagged ArfGAP1. LRRK2 but not parkin interacts directly with ArfGAP1 (Figure S1). To identify the domain of LRRK2 responsible for the interaction with ArfGAP1, co-immunoprecipitation assays were conducted in cells co-expressing ArfGAP1-YFP and various deletion mutants of LRRK2 (Figure 1C). Following immunoprecipitation of LRRK2 deletion mutants we unexpectedly find that ArfGAP1 interacts specifically with N-terminal residues 480–895 of LRRK2, a region containing LRRK2-specific repeats (residues 1–660), armadillo repeats (residues 180–660), and putative ankyrin repeats (residues 690–860) known to mediate protein-protein interactions, rather than with the Roc GTPase domain of LRRK2. We could confirm that our LRRK2 protein fragment encompassing the GTPase domain (F3, residues 895–1503) is correctly folded and functional since it could bind efficiently to GTP-sepharose in pull-down assays and could interact with full-length GFP-tagged LRRK2 by co-immunoprecipitation indicative of dimer formation (Figure S2). To assess the specificity of the LRRK2/ArfGAP1 interaction, the potential interaction of ArfGAP1 with the PD-associated protein α-synuclein was assessed in HEK-293T cells. However, ArfGAP1-YFP fails to interact with myc-tagged α-synuclein (Figure 1D). We next sought to verify the interaction of LRRK2 and ArfGAP1 *in vivo*. LRRK2 interacts with ArfGAP1 in brain extracts derived from wild-type mice following immunoprecipitation with a LRRK2-specific monoclonal antibody (MJFF-2/c41-2), whereas ArfGAP1 is not immunoprecipitated in extracts derived from LRRK2 knockout mice (Figure 1E). ArfGAP1 also interacts with LRRK2 in wild-type mouse brain following immunoprecipitation with an ArfGAP1-specific rabbit antibody but not with a non-specific rabbit IgG control (Figure 1F). Collectively, our data demonstrate that LRRK2 interacts directly with ArfGAP1 *in vitro*, in cultured cells and *in vivo* in brain tissue.

### Co-localization of LRRK2 with ArfGAP1 in the cytoplasm and at Golgi membranes of mammalian cells and neurons

**Figure 2:**
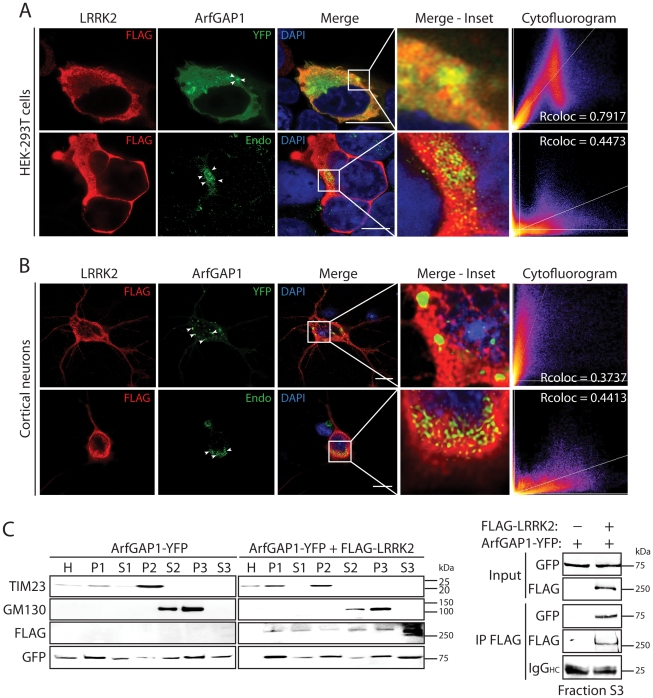
Co-localization of LRRK2 and ArfGAP1 in mammalian cells and neurons. (A) Confocal fluorescence microscopy reveals the co-localization of FLAG-tagged human LRRK2 with YFP-tagged and endogenous ArfGAP1 within the cytoplasm and at Golgi membranes of HEK-293T cells. ArfGAP1-YFP expression induces the dispersal of the Golgi complex and localizes to Golgi-derived vesicles and within the cytoplasm, whereas endogenous ArfGAP1 localizes to the intact Golgi complex (*arrowheads*) and at lower levels within the cytoplasm. Scale bars: 10 µm. (B) Similar co-localization of FLAG-LRRK2 with ArfGAP1-YFP or endogenous ArfGAP1 within the cytoplasm and at Golgi membranes (*arrowheads*) in rat primary cortical neurons. Cytofluorograms and co-localization coefficients (Rcoloc) reveal the extent of co-localization between LRRK2 and ArfGAP1 fluorescence signals with strongest correlation between LRRK2 and ArfGAP1-YFP in HEK-293T cells, and LRRK2 and endogenous ArfGAP1 in cortical neurons. Confocal images are taken from single z-plane at 0.1 µm thickness. Images are representative of at least three independent transfection experiments. Scale bars: 10 µm. (C) Subcellular fractionation of HEK-293T cells expressing ArfGAP1-YFP and FLAG-LRRK2. LRRK2 and ArfGAP1 are detected together in multiple subcellular fractions including soluble cytosolic (S1-S3), heavy (P2, mitochondria/TIM23) and light (P3, Golgi/GM130) membrane fractions (*left panels*). ArfGAP1-YFP interacts with FLAG-LRRK2 in the membrane-deficient soluble cytosolic fraction (S3) following IP with anti-FLAG antibody (*right panel*). Molecular mass markers are indicated in kilodalton (kDa). Blots are representative of at least two independent experiments.

To further characterize the interaction of LRRK2 with ArfGAP1, and where in cells this might occur, we assessed the co-localization of both proteins in mammalian cells by confocal microscopy. ArfGAP1 normally localizes to the Golgi complex but also cycles from the Golgi to the cytoplasm following Arf1 inactivation [39], [40]. The overexpression of ArfGAP1 leads to excessive Arf1 inactivation and promotes the fusion of the Golgi with the ER resulting in the dispersal of Golgi-derived vesicles with the Golgi complex ceasing to exist as a discrete organelle [40]. Co-expression of FLAG-LRRK2 and ArfGAP1-YFP in HEK-293T cells reveals the localization of ArfGAP1 to Golgi-derived vesicles and the cytoplasm, with LRRK2 mostly co-localizing with the cytoplasmic portion of ArfGAP1 but also at vesicle membranes (Figure 2A). FLAG-LRRK2 also co-localizes with endogenous ArfGAP1 within the cytoplasm and at the membrane surface of the intact Golgi complex in HEK-293T cells (Figure 2A). We also performed similar co-localization experiments in rat primary cortical neurons. Exogenous FLAG-LRRK2 co-localizes with exogenous ArfGAP1-YFP and endogenous ArfGAP1 within the cytoplasm and at the membrane surface of either Golgi-derived vesicles or the intact Golgi complex, respectively, within neuronal soma (Figure 2B). It is not possible to reliably assess co-localization of endogenous LRRK2 and ArfGAP1 in HEK-293T cells or primary neurons with currently available antibodies. To determine whether LRRK2 and ArfGAP1 are capable of interacting together within the cytoplasm, we prepared extracts from transfected HEK-293T cells by subcellular fractionation that are devoid of membranous organelles including Golgi membranes (soluble S3 fraction, 100K) followed by co-immunoprecipitation assays. FLAG-LRRK2 robustly interacts with ArfGAP1-YFP in Golgi-free cell extracts (Figure 2C) thus confirming that LRRK2 and ArfGAP1 can interact within the cytoplasm and do not critically require Golgi membranes for their interaction. Furthermore, FLAG-LRRK2 and ArfGAP1-YFP are shown to co-associate in membranous P2 (heavy membranes i.e. mitochondria) and P3 (light membranes i.e. Golgi) or soluble cytosolic (S1–S3) subcellular fractions in HEK-293T cells (Figure 2C). Therefore, ArfGAP1 exists within both membrane-associated and soluble cytosolic subcellular compartments of mammalian cells supporting its dynamic interaction with membranes.

**Figure 3:**
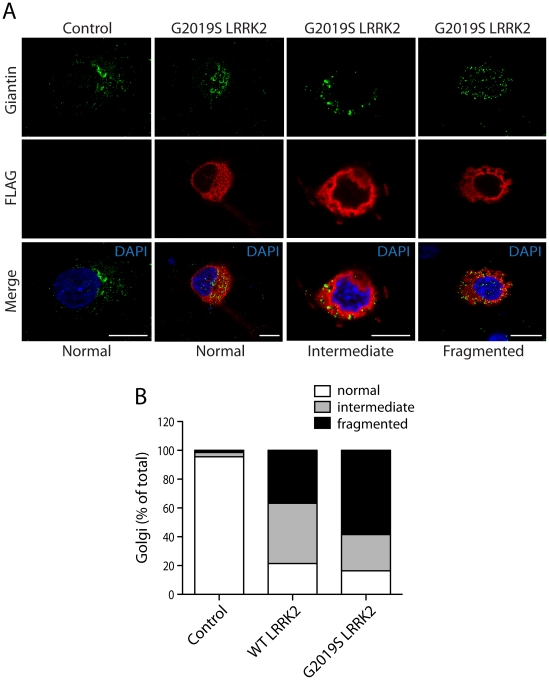
LRRK2 expression induces Golgi fragmentation in cortical neurons. (A) Rat primary cortical neurons were transfected at DIV 3 with FLAG-LRRK2 variants or control empty plasmids. Cultures were fixed at DIV 6 and subjected to immunocytochemistry with antibodies to FLAG and the Golgi membrane marker, Giantin. Confocal fluorescence microscopy reveals the effects of G2019S LRRK2 expression on Golgi morphology, resulting in a normal, partially fragmented (intermediate) or fully fragmented Golgi complex. (B) Quantitative analysis of Golgi fragmentation induced by WT or G2019S LRRK2 expression in cortical neurons. Golgi were subclassified as normal, intermediate or fragmented in FLAG-positive neurons expressing WT LRRK2 (*n* = 359) or G2019S LRRK2 (*n* = 238) compared to control neurons (empty plasmid; *n* = 498). Golgi subclasses were expressed as a percent of the total number of Golgi per condition from three independent cultures.

We could confirm the localization of endogenous ArfGAP1 to the Golgi complex in HEK-293T cells, and the variable localization of exogenous ArfGAP1-YFP to either the intact Golgi complex or Golgi-derived vesicles depending upon its degree of overexpression (Figure S3A), as previously reported [40]. ArfGAP1-YFP overexpression frequently induces the dispersal of the intact Golgi complex with the appearance of ArfGAP1-positive Golgi-derived vesicles that are devoid of Golgi markers (Figure S3A). We could further confirm the localization of endogenous ArfGAP1 to the Golgi complex and exogenous ArfGAP1-YFP to Golgi-derived vesicles in primary cortical neurons (Figure S3B). Endogenous LRRK2 and exogenous FLAG-LRRK2 also localize to a small extent with the Golgi complex in cortical neurons with LRRK2-positive punctate structures decorating Golgi membranes (Figure S3B). To determine whether the overexpression of LRRK2 influences the integrity of the Golgi complex, similar to ArfGAP1 overexpression, the effects of exogenous FLAG-LRRK2 on Golgi morphology was assessed in primary cortical neurons (Figure 3). The overexpression of FLAG-LRRK2 in cortical neurons induces the partial and complete fragmentation of the Golgi complex with the familial G2019S mutant LRRK2 producing greater fragmentation than wild-type (WT) LRRK2 (Figure 3). Therefore, one aspect of LRRK2-induced neuronal toxicity may relate to disruption of the normal Golgi complex, and potentially suggests that LRRK2 and ArfGAP1 act together in a common pathway to regulate Golgi morphology and function. Together, our data reveal the co-localization of LRRK2 with exogenous or endogenous ArfGAP1 occurring within the cytoplasm and at Golgi membranes. Furthermore, the overexpression of LRRK2 or ArfGAP1 commonly promotes the fragmentation and dispersal of the Golgi complex.

### PD–associated and functional mutations in LRRK2 modulate the interaction with ArfGAP1

**Figure 4:**
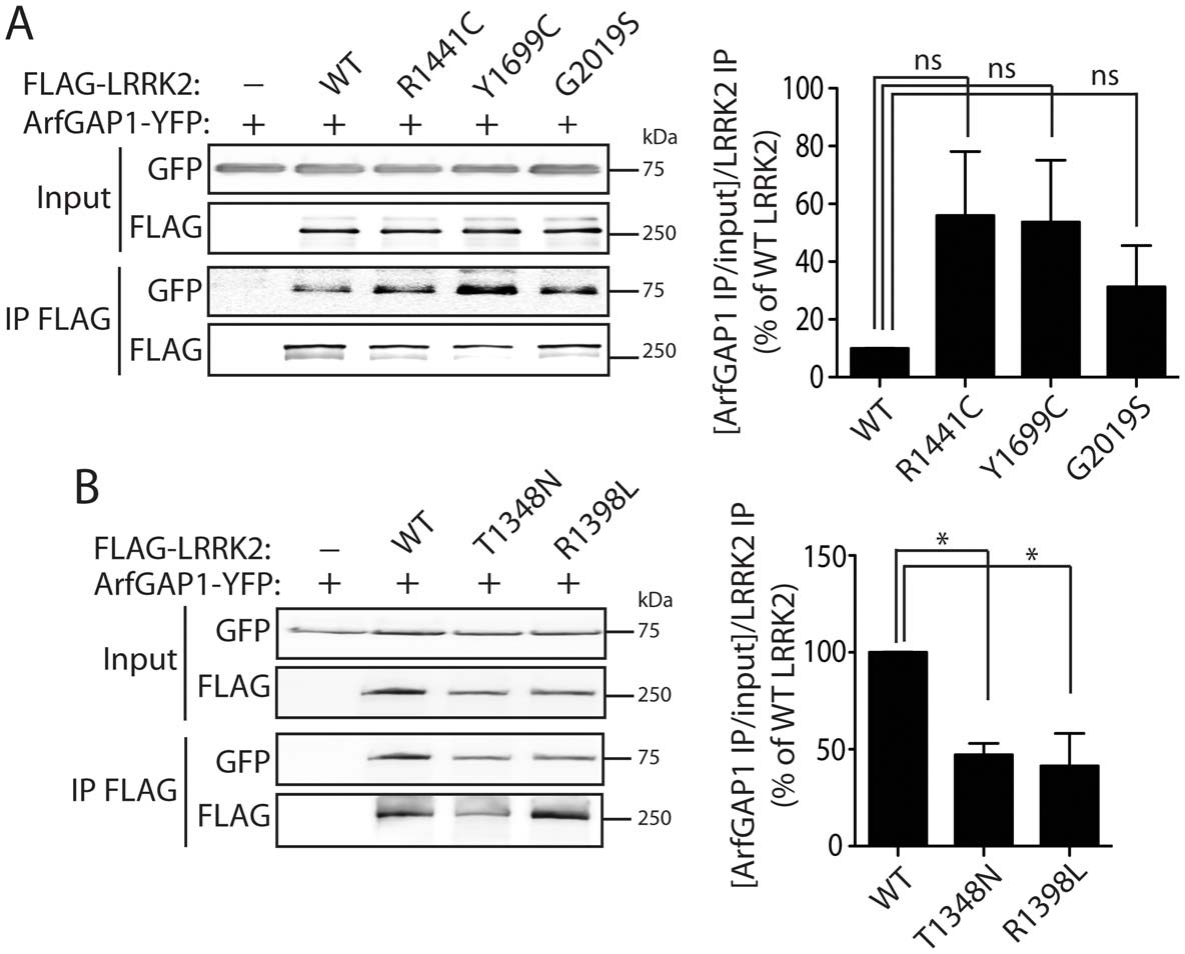
Familial PD–associated and functional mutations in LRRK2 modulate the interaction with ArfGAP1. (A) Differential interaction of YFP-tagged ArfGAP1 with WT and PD-associated mutant forms of FLAG-tagged human LRRK2 following IP with anti-FLAG antibody from HEK-293T cells. Densitometric analysis reveals a non-significant increase in the interaction of ArfGAP1 with R1441C, Y1699C and G2019S LRRK2 compared to WT LRRK2. (B) Functional GTPase mutations in FLAG-LRRK2 impair the interaction with ArfGAP1-YFP following IP with anti-FLAG antibody from HEK-293T cells. Densitometry reveals a significant reduction of the interaction of ArfGAP1 with T1348N and R1398L LRRK2 compared to WT LRRK2. Data represent the level of interaction of ArfGAP1 with LRRK2 expressed as a percent of the interaction with WT LRRK2. The levels of ArfGAP1 IP were first normalized to ArfGAP1 input levels, and then further normalized to LRRK2 IP levels. Bars represent the mean ± SEM (*n* = 3 experiments). *P<0.05 compared to WT LRRK2 by one-way ANOVA with Newman-Keuls post-hoc analysis. *ns*, non-significant.

To investigate the impact of PD-associated familial mutations on the interaction of LRRK2 with ArfGAP1, we conducted co-immunoprecipitation analysis in HEK-293T cells expressing ArfGAP1-YFP and FLAG-LRRK2 variants. Compared to WT LRRK2, the familial mutants R1441C, Y1699C and G2019S commonly induce a marked yet non-significant increase in the interaction of LRRK2 with ArfGAP1 (Figure 4A). The R1441C and Y1699C variants are localized to the Roc GTPase and COR domains, respectively, and have been shown to modestly impair GTP hydrolysis and lead to an increase in the steady-state levels of GTP-bound LRRK2 [17], [20], [27]. Accordingly, we assessed the effects of functional non-pathogenic mutations known to modulate the GTPase activity of LRRK2. We employed a T1348N variant in the P-loop which abolishes GDP/GTP binding and impairs GTP hydrolysis [16], and a R1398L variant in the Switch II catalytic region which enhances GTP hydrolysis and reduces GTP binding [27]. The T1348N and R1398L mutations significantly impair the interaction of LRRK2 with ArfGAP1 compared to WT LRRK2 (Figure 4B). Therefore, ArfGAP1 preferentially interacts with GTP-bound LRRK2 rather than GDP-bound or guanine nucleotide-deficient LRRK2, consistent with GAP proteins which exhibit higher affinity for GTP-bound GTPases. Taken together, our data demonstrate that familial and functional mutations which impair GTP hydrolysis and increase the levels of GTP-bound LRRK2 increase the interaction of LRRK2 with ArfGAP1.

### Distribution of ArfGAP1 in the mammalian brain

**Figure 5:**
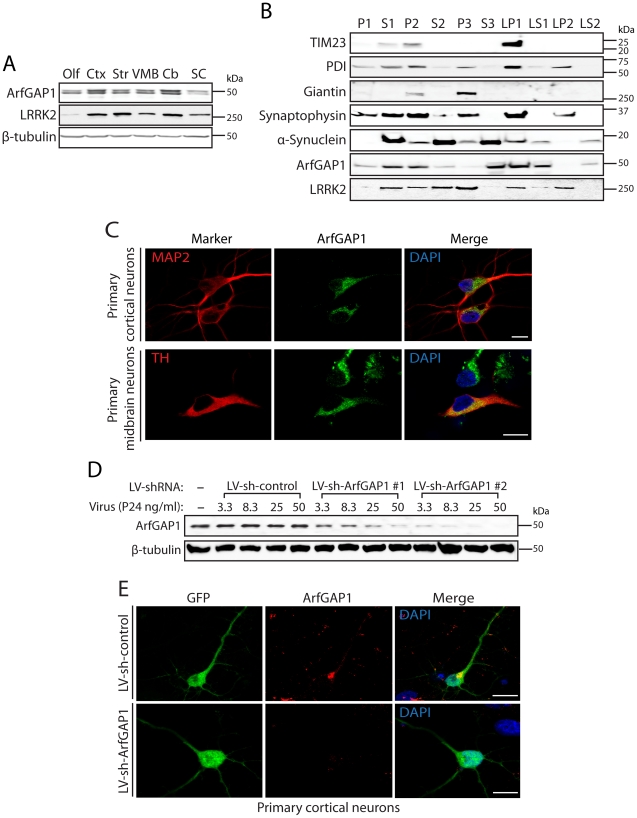
ArfGAP1 is expressed in the mammalian brain. (A) Western blot analysis of ArfGAP1 protein levels in mouse brain, with highest levels detected in cerebral cortex (*Ctx*) and cerebellum (*Cb*), moderate levels in striatum (*Str*) and ventral midbrain (*VMB*), and lowest levels in olfactory bulb (*Olf*) and spinal cord (*SC*). LRRK2 is detected at highest levels in striatum, cortex and cerebellum. β-tubulin indicates equivalent protein loading. (B) Subcellular fractionation of mouse whole brain tissue. ArfGAP1 is broadly detected with particular enrichment in membrane-associated fractions including the heavy (P2) and synaptosomal (LP1) membrane fractions, and soluble cytosolic (S1 and S3) and synaptosomal cytosolic (LS1) fractions. LRRK2 (MJFF-2/c41-2) is also broadly detected with enrichment in membrane-associated fractions, including heavy (P2) and light (P3) membrane, synaptosomal (LP1) and synaptic vesicle (LP2) fractions, in addition to soluble cytosolic (S1 and S2) and synaptosomal cytosolic (LS1) fractions. ArfGAP1 and LRRK2 co-associate in the S1, P2, S2, LP1 and LS1 fractions. The distribution of marker proteins demonstrates the enrichment of mitochondria/heavy membranes (TIM23; P2 and LP1), endoplasmic reticulum (PDI; P2, P3, LP1 and LP2), Golgi (Giantin; P2 and P3), synaptosomes/synaptic vesicles (synaptophysin 1; P2, P3, LP1 and LP2) and synaptosomal/synaptic vesicle cytosolic (α-synuclein; LS1 and LS2). (C) Confocal fluorescence microscopy reveals the localization of endogenous ArfGAP1 to the Golgi complex and cytoplasm of MAP2-positive cortical neurons and tyrosine hydroxylase (TH)-positive dopaminergic neurons in rat primary cortical and midbrain cultures, respectively. (D) Validation of short hairpin RNA (shRNA)-mediated silencing of endogenous ArfGAP1 expression in rat primary cortical neuronal cultures. Lentiviral vectors expressing non-silencing shRNA (LV-sh-control) or ArfGAP1-specific shRNAs (LV-sh-ArfGAP1 #1 and LV-sh-ArfGAP1 #2) were used to infect primary neurons at increasing viral doses ranging from 3.3 to 50 ng of P24 antigen per ml of media. Western blot analysis of neuronal extracts reveals the viral dose-dependent knockdown of ArfGAP1 with ArfGAP1-specific shRNAs but not control shRNA. β-tubulin indicates equivalent protein loading. (E) Silencing of endogenous ArfGAP1 expression in GFP-labeled cortical neurons with lentiviral-shRNA vectors revealed by confocal fluorescence microscopy with a rabbit anti-ArfGAP1 antibody. Molecular mass markers are indicated in kilodalton (kDa). Scale bars: 10 µm.

LRRK2 is widely distributed throughout the mammalian brain where it localizes to intracellular vesicular and membranous structures within neurons. These structures include acidic vesicles (i.e. lysosomes, endosomes, Golgi-derived vesicles, microtubule-associated vesicles and multivesicular bodies), mitochondria, the Golgi complex, the endoplasmic reticulum (ER) and lipid rafts [44], [45], [46]. However, the distribution of ArfGAP1 within the mammalian brain has not been described in detail but is enriched in the brain relative to other tissues [47]. To further assess the physiological relevance of the LRRK2/ArfGAP1 interaction, we explored the localization of ArfGAP1 in the mammalian brain. Western blot analysis of ArfGAP1 expression in distinct anatomic regions of mouse brain reveals a broad expression profile with highest levels detected in the cerebral cortex and cerebellum, moderate levels in the striatum and ventral midbrain, and lowest levels in the olfactory bulb and spinal cord (Figure 5A). To further explore the localization of ArfGAP1, we conducted subcellular fractionation of mouse brain tissue. ArfGAP1 is broadly detected in a number of subcellular fractions with particular enrichment in soluble cytosolic (S1 and S3), heavy membrane (P2, i.e. mitochondria and crude synaptosomes), synaptosomal membrane (LP1) and synaptosomal/synaptic vesicle cytosolic (LS1 and LS2) fractions (Figure 5B). For comparison, LRRK2 is enriched in heavy (P2) and light (P3, i.e. ER and Golgi) membrane, synaptosomal membrane (LP1) and synaptic vesicle-enriched (LP2) fractions (Figure 5B). Importantly, ArfGAP1 and LRRK2 co-associate in heavy membrane (P2), synaptosomal membrane (LP1), and synaptosomal cytosolic (LS1) fractions as well as other soluble fractions (S1 and S2) (Figure 5B). Notably, ArfGAP1 is detected in both membrane (P2 and LP1) and soluble cytosolic (S1–S3, LS1 and LS2) fractions in brain tissue supporting its dynamic and transient interaction with intracellular membranes such as the Golgi complex. Next, we explored the localization of endogenous ArfGAP1 in primary neuronal cultures derived from the cortex or ventral midbrain of post-natal rats. Confocal fluorescence microscopy demonstrates the localization of endogenous ArfGAP1 to the Golgi complex and cytoplasm of microtubule-associated protein 2 (MAP2)-labeled neurons in cortical cultures and tyrosine hydroxylase (TH)-labeled dopaminergic neurons in midbrain cultures (Figure 5C). To confirm the specificity of our ArfGAP1 antibody we developed lentiviral vectors expressing short hairpin RNAs (shRNAs) for efficient silencing of ArfGAP1 expression. Primary cortical neurons were infected with lentiviral-shRNAs and endogenous ArfGAP1 expression was monitored by Western blot analysis of neuronal extracts or by confocal microscopy. Two independent shRNAs directed against rat ArfGAP1 induce the viral dose-dependent knockdown of endogenous ArfGAP1 levels compared to a non-silencing control shRNA in cortical neurons (Figure 5D). We also demonstrate the loss of ArfGAP1-positive fluorescent signal in GFP-labeled cortical neurons following shRNA-mediated knockdown of endogenous ArfGAP1 compared to a control shRNA (Figure 5E). Thus, this antibody specifically labels endogenous ArfGAP1 in cortical neurons, and furthermore validates the efficient knockdown of endogenous ArfGAP1 in cortical neurons using lentiviral-shRNA vectors. Finally, two ArfGAP1-specific antibodies were employed to assess the distribution of ArfGAP1 in intact tissue sections from mouse brain. However, both antibodies were not suitable for specifically labeling ArfGAP1 in brain sections (data not shown). Collectively, our data demonstrate that ArfGAP1 is expressed throughout the mammalian brain including within cortical and midbrain dopaminergic neuronal populations where it localizes to both cytosolic and membrane-associated subcellular compartments.

### ArfGAP1 enhances the GTPase activity of LRRK2

**Figure 6:**
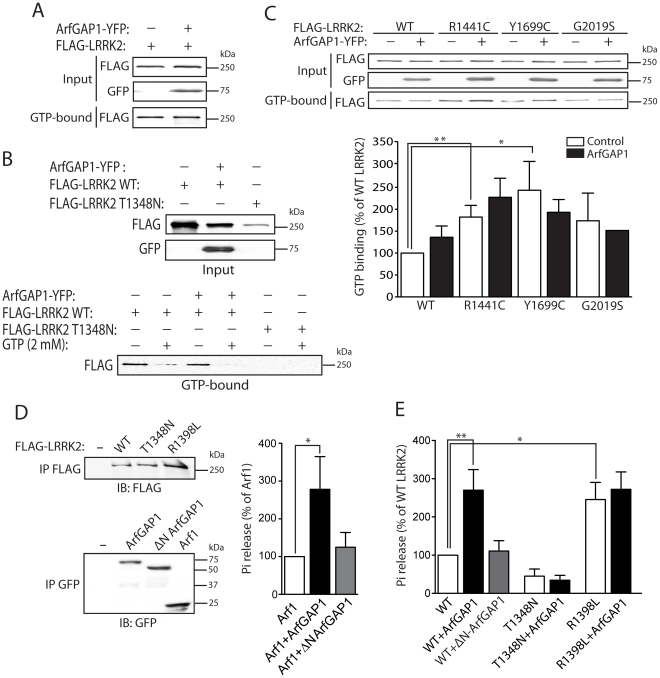
ArfGAP1 enhances the GTP hydrolysis activity of LRRK2. (A) ArfGAP1-YFP expression fails to influence the steady-state levels of FLAG-tagged WT LRRK2 bound to GTP following pull-down assays with GTP-sepharose from HEK-293T cells. (B) Confirmation of the specificity of LRRK2 GTP binding is indicated by negligible binding of the GDP/GTP binding-deficient LRRK2 mutant, T1348N. Competition with an excess of free GTP (2 mM) abolishes binding of WT LRRK2 to GTP-sepharose in the presence or absence of ArfGAP1. Each input lysate (*top panel*) was split equally and used for GTP pull-down assays in the presence or absence of free GTP (*lower panel*), as indicated. (C) ArfGAP1-YFP expression fails to influence the steady-state levels of GTP-bound WT and PD-associated mutant LRRK2. Densitometric analysis reveals a significant increase in GTP binding of the R1441C and Y1699C LRRK2 variants compared to WT LRRK2, independent of ArfGAP1 expression. Data represent the level of LRRK2 GTP binding expressed as a percent of WT LRRK2 levels. GTP-bound LRRK2 levels were normalized to LRRK2 input levels. Bars represent the mean ± SEM (*n* = 3 experiments). *P<0.05 and **P<0.01 compared to WT LRRK2 by one-way ANOVA with Newman-Keuls post-hoc analysis. (D) GTP hydrolysis activity of Arf1 in the presence or absence of full-length ArfGAP1 was determined by measuring the concentration of free phosphate (P_i_) released from GTP. WT ArfGAP1 enhances Arf1-mediated GTP hydrolysis whereas ΔN ArfGAP1 has negligible effects on Arf1. The input levels of immunopurified Arf1-CFP and ArfGAP1-YFP variant were assessed by Western blot analysis with anti-GFP antibody (*lower panel*). (E) GTP hydrolysis activity of WT LRRK2 is significantly enhanced by WT ArfGAP1 but not by ΔN ArfGAP1. Guanine nucleotide-deficient T1348N LRRK2 exhibits impaired GTP hydrolysis activity, whereas the GTPase-hyperactive R1398L LRRK2 exhibits enhanced activity compared to WT LRRK2. The input levels of immunopurified FLAG-LRRK2 variants were assessed by Western blot analysis with anti-FLAG antibody (E, *upper panel*), quantified by densitometry, and used for normalization of P_i_ release between LRRK2 variants. GTP hydrolysis activity for Arf1 (D) or LRRK2 (E) is expressed as P_i_ release as a percent of Arf1 or WT LRRK2 activity alone, as indicated. Bars represent the mean ± SEM (*n* = 5 experiments). *P<0.05 and **P<0.01 compared to Arf1 (D) or WT LRRK2 (E) by one-way ANOVA with Newman-Keuls post-hoc analysis.

ArfGAP1 functions as a GAP protein to promote the GTP hydrolysis of the small GTPase Arf1 [39]. Additional GTPases regulated by ArfGAP1 have not yet been identified. To determine whether the interaction with ArfGAP1 may serve to regulate the GTPase activity of LRRK2, we explored the effects of ArfGAP1 on LRRK2 GTP binding and hydrolysis. To monitor the effects of ArfGAP1 on the steady-state levels of GTP-bound LRRK2, we conducted pull-down assays using GTP-sepharose from HEK-293T cell extracts expressing FLAG-LRRK2 with or without ArfGAP1-YFP. ArfGAP1 expression fails to influence the levels of GTP-bound LRRK2 (Figure 6A). Control experiments confirm the specificity of LRRK2 for binding to immobilized GTP as revealed by a reduction in GTP-bound LRRK2 following competition with an excess of free GTP, or by using a T1348N variant of LRRK2 that abolishes binding to guanine nucleotides (Figure 6B). Next, we compared the effects of ArfGAP1 on the steady-state levels of GTP-bound LRRK2 harboring familial PD-associated mutations. ArfGAP1 expression fails to appreciably influence the levels of GTP-bound WT or mutant LRRK2, whereas the R1441C and Y1699C mutations significantly enhance the levels of GTP-bound LRRK2 independent of ArfGAP1 expression levels (Figure 6C), as previously reported [20]. The increased levels of GTP-bound LRRK2 for these familial mutations (i.e. R1441C and Y1699C) correlates with their increased interaction with ArfGAP1 (refer to Figure 4A), further suggesting a preference of ArfGAP1 for interacting with GTP-bound LRRK2.

To assess the impact of ArfGAP1 on LRRK2 GTPase activity we performed a well-established *in vitro* assay with immunopurified proteins to monitor LRRK2-mediated GTP hydrolysis by measuring the release of free γ-phosphate produced by hydrolysis of GTP to GDP [27]. To initially determine whether ArfGAP1 is functional in these assays, the effects of ArfGAP1 on Arf1-mediated GTP hydrolysis were assessed. As expected, ArfGAP1 enhances Arf1 GTP hydrolysis by >2.5-fold whereas a ΔN-ArfGAP1 deletion mutant with negligible GAP activity fails to modify Arf1 GTP hydrolysis (Figure 6D). Thus, ArfGAP1 is functional in this GTPase assay consistent with the functional effect of ArfGAP1 overexpression on Golgi dispersal (refer to Figure 2 and Figure S3). Next, we assessed the impact of ArfGAP1 on LRRK2-mediated GTP hydrolysis. Similar to its effect on Arf1, ArfGAP1 enhances the GTP hydrolysis of WT LRRK2 by >2.5-fold whereas ΔN-ArfGAP1 has no effect (Figure 6E). The GDP/GTP-binding-deficient T1348N LRRK2 variant exhibits markedly diminished GTP hydrolysis activity that does not increase upon addition of ArfGAP1 (Figure 6E). Furthermore, a GTPase-hyperactive R1398L LRRK2 variant exhibits a >2.5-fold increase in GTP hydrolysis, compared to WT LRRK2, which is not further enhanced by addition of ArfGAP1 (Figure 6E). The lack of effect of ArfGAP1 on R1398L LRRK2 suggests that this mutant may already possess maximal GTPase activity in this assay, and is comparable to the effect of ArfGAP1 on WT LRRK2. Collectively, our data identify ArfGAP1 as a novel GAP protein for modulating the GTPase activity of LRRK2.

### ArfGAP1 is directly phosphorylated by LRRK2 and enhances LRRK2 kinase activity

**Figure 7:**
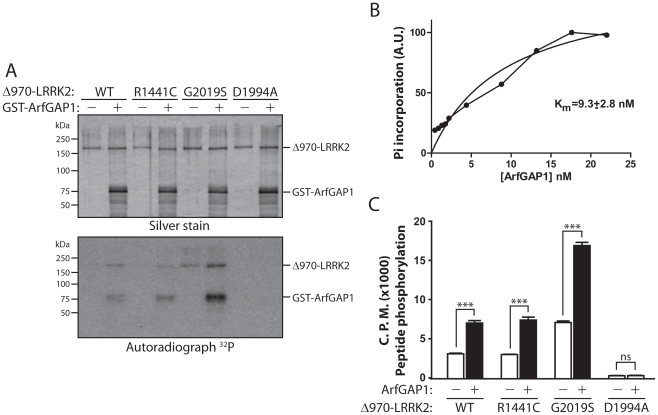
LRRK2 phosphorylates ArfGAP1, and ArfGAP1 enhances LRRK2 kinase activity. (A) *In vitro* kinase assay with [^32^P]-γ-ATP, recombinant WT, R1441C, G2019S or D1994A human LRRK2 (Δ970, residues 970–2527) and GST-tagged ArfGAP1. Silver-stained SDS-PAGE gels indicate equal loading of ArfGAP1 and LRRK2 proteins in each condition. Autoradiographs show the LRRK2-dependent phosphorylation of ArfGAP1, with enhanced phosphorylation by G2019S LRRK2 compared to WT protein. LRRK2 autophosphorylation is also detected in this assay. Molecular mass markers are indicated in kilodaltons (kDa). (B) Michaelis-Menten kinetics of ArfGAP1 as a LRRK2 substrate. Reactions were run for 30 min in the presence of 2 nM WT-LRRK2 enzyme and the indicated concentration of ArfGAP1. Incorporation of phosphate into ArfGAP1 was determined by SDS-PAGE followed by densitometry from a phosphoscreen. (C) *In vitro* radioactive kinase assay using recombinant Δ970-LRRK2 variants and LRRKtide peptide as a LRRK2 substrate. WT, R1441C and G2019S LRRK2 phosphorylate LRRKtide at levels correlating with LRRK2 autophosphorylation, whereas kinase-dead D1994A shows minimal activity towards LRRKtide. ArfGAP1 significantly enhances LRRKtide phosphorylation by LRRK2 variants. Data represent the level of LRRKtide phosphorylation expressed as ^32^P incorporation in radioactive counts per minute (CPM). Bars represent the mean ± SEM (*n* = 4 experiments). ***P<0.001 comparing the absence or presence of ArfGAP1 for each LRRK2 variant by one-way ANOVA with Newman-Keuls post-hoc analysis. *ns*, non-significant.

To determine whether there is a reciprocal relationship between LRRK2 and ArfGAP1 enzymatic activities, we assessed whether ArfGAP1 regulates LRRK2 kinase activity and whether ArfGAP1 is a substrate of LRRK2-mediated phosphorylation. *In vitro* kinase assays using [^32^P]-γ-ATP with recombinant LRRK2 (residues 970–2527) and full-length GST-ArfGAP1 proteins reveal that WT, R1441C and G2019S LRRK2 can robustly phosphorylate ArfGAP1 (Figure 7A and 7B). A kinase-dead LRRK2 variant (D1994A) has negligible effects on ArfGAP1 phosphorylation (Figure 7A). Similar results were obtained using immunopurified full-length LRRK2 variants for *in vitro* kinase assays (Figure S4). Several LRRK2 kinase substrates have previously been proposed, but these have generally shown weak phosphorylation compared to intrinsic LRRK2 autophosphorylation [11], [12], [13], [15], [48]. In contrast to previously published substrates, natively folded ArfGAP1 demonstrates a K(m) of 9.3±2.8 nM in kinase reactions containing 2 nM LRRK2 enzyme, suggesting efficient phosphorylation of ArfGAP1 by LRRK2 (Figure 7B). Therefore, ArfGAP1 represents a novel, robust substrate of LRRK2-mediated phosphorylation *in vitro*.

Unexpectedly, we noticed that inclusion of ArfGAP1 enhances LRRK2 autophosphorylation (Figure 7A). Inclusion of a peptide substrate for LRRK2 in the same reaction demonstrates that ArfGAP1 enhances the kinase activity of WT, R1441C and G2019S LRRK2 by >2-fold (Figure 7C). Our data suggest that modulation of GTPase activity by ArfGAP1 enhances the kinase activity of LRRK2.

### Silencing of ArfGAP1 expression rescues G2019S LRRK2-induced neurite shortening

**Figure 8:**
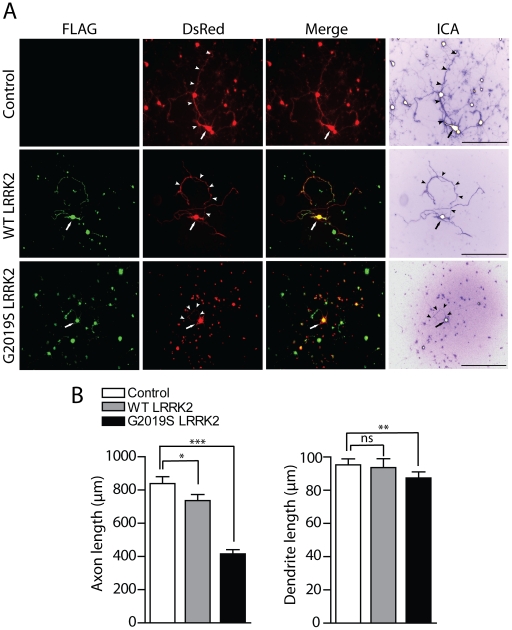
Effects of LRRK2 expression on neurite length of cortical neurons. (A) Rat primary cortical neurons were transfected at DIV 3 with FLAG-LRRK2 variants and DsRed constructs at a DNA molar ratio of 10∶1. Cultures were fixed at DIV 6. Fluorescent microscopic images reveal the co-labeling of cortical neurons with FLAG-LRRK2 (WT or G2019S) and DsRed. DsRed images were pseudo-colored with ICA to improve the contrast of neuritic processes for length measurements. Neuronal soma (*arrows*) and axonal processes (*arrowheads*) are indicated. Scale bars: 400 µm. (B) Analysis of the length of DsRed-positive neurites reveals a significant shortening of axons and dendrites due to G2019S LRRK2 expression, with smaller effects of WT LRRK2 on axons only, compared to DsRed alone (control). Bars represent mean (± SEM) length of axons or dendrites in µm from 90–120 DsRed-positive neurons from at least three independent experiments/cultures. *P<0.05, **P<0.01 and ***P<0.001 compared to control (DsRed alone) by one-way ANOVA with Newman-Keuls post-hoc analysis. *ns*, non-significant.

The overexpression of familial LRRK2 mutations promotes neuronal toxicity and cell death in primary cultures in a manner dependent upon GTPase and kinase activity [9], [19], [20], [30]. LRRK2 has also been shown to robustly regulate the morphology of neuronal processes with overexpression of familial LRRK2 mutants (i.e. G2019S and I2020T) reducing neurite length and complexity and LRRK2 deletion or knockdown producing opposing effects [35], [36], [38], [49]. As GTPase activity is required for the neurotoxic effects of WT and mutant LRRK2, we explored the effects of ArfGAP1 expression on LRRK2-induced neurite shortening. First, we assessed the effects of LRRK2 overexpression on neurite length in primary cortical and midbrain dopaminergic neurons in order to develop a robust quantitative assay of neurite shortening. Primary cortical and midbrain cultures were transiently co-transfected with FLAG-LRRK2 variants and DsRed-Max (cortical) or GFP (midbrain) at a DNA molar ratio of 10∶1 to morphologically label transfected neurons (Figure 8A and Figure S5). At 3 days post-transfection, the length of DsRed-positive or GFP-positive neurites were determined and assigned as dendritic or axonal processes. For cortical cultures, neurite analysis was restricted to the MAP2-positive neuronal population, whereas for midbrain cultures, neurite analysis was conducted on TH-positive dopaminergic neurons. The overexpression of G2019S LRRK2 leads to a robust shortening of axonal processes from cortical neurons with a smaller effect of WT LRRK2, compared to neurons expressing DsRed alone (Figure 8A and 8B). In contrast, G2019S LRRK2 has only a modest effect on the length of dendritic processes from cortical neurons in this assay, whereas the effects of WT LRRK2 are negligible (Figure 8A and 8B). The effects of LRRK2 overexpression on the length of dopaminergic neuronal processes were less robust with G2019S LRRK2 producing a negligible effect on axonal length but unexpectedly a small significant increase in the length of dendritic processes compared to neurons expressing GFP alone (Figure S5). Therefore, we decided to focus our attention on primary cortical neurons as a robust model for assessing LRRK2-induced neurite shortening.

To confirm that G2019S LRRK2 overexpression specifically influences the length of axonal processes, cortical neurons were co-transfected as described above with FLAG-LRRK2 and the axonal marker GFP-tagged tau (*MAPT*) at a DNA molar ratio of 10∶1 to morphologically label axonal processes of transfected neurons (Figure S6A). The overexpression of G2019S LRRK2 leads to a robust shortening of GFP-tau-positive axonal processes from cortical neurons compared to neurons expressing GFP-tau alone (Figure S6B). To determine whether the overexpression of LRRK2 also induces neuronal cell death in our primary cortical culture model, cultures were co-transfected with FLAG-LRRK2 variants and GFP constructs at a 10∶1 molar ratio at DIV 11, fixed at DIV 14, and subjected to TUNEL labeling to detect apoptotic cells. The overexpression of WT or G2019S LRRK2 does not appreciably induce the apoptotic cell death of cortical neurons compared to control neurons expressing GFP alone (Figure S6C). Therefore, G2019S LRRK2 induces robust neurite shortening independent of apoptotic cell death in this rat primary cortical neuronal model.

**Figure 9:**
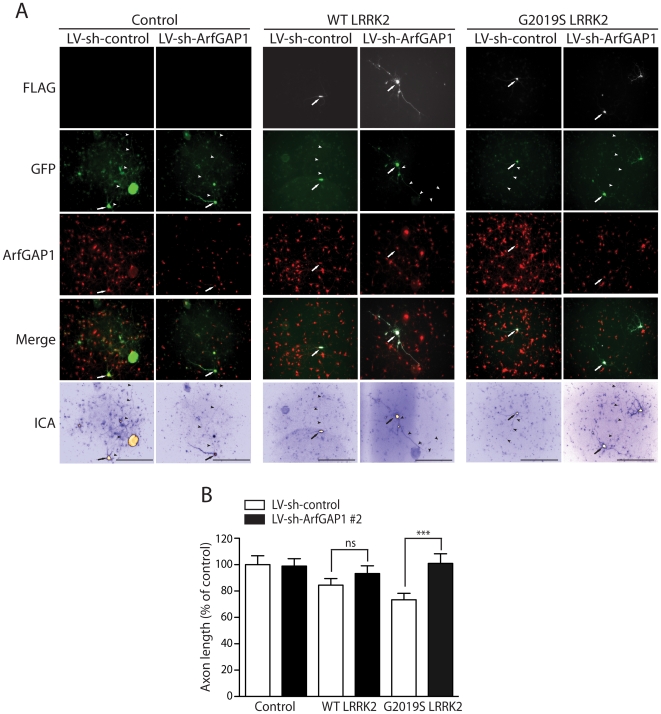
Silencing of ArfGAP1 expression rescues G2019S LRRK2-induced neurite shortening. (A) Primary cortical neurons were infected with lentiviral vectors expressing shRNAs (non-silencing control or ArfGAP1-specific) at DIV 2, subsequently transfected with FLAG-LRRK2 (WT or G2019S) and GFP constructs at a 10∶1 molar ratio at DIV 3, and fixed at DIV 6. Fluorescent microscopic images reveal the co-labeling of cortical neurons with FLAG-LRRK2 and GFP. The knockdown of endogenous ArfGAP1 fluorescence signal is also shown following lentiviral expression of ArfGAP1-specific shRNA (LV-sh-ArfGAP1 #2) compared to control shRNA (LV-sh-control). GFP images were pseudo-colored with ICA for neurite length measurements. Neuronal soma (*arrows*) and axonal processes (*arrowheads*) are indicated. Scale bars: 400 µm. (B) Analysis of GFP-positive axonal processes reveals a robust shortening of axons induced by G2019S LRRK2 expression, and a smaller effect induced by WT LRRK2, compared to GFP alone (control). Knockdown of ArfGAP1 with lentiviral-shRNA vectors produces a complete rescue of G2019S LRRK2-induced axon shortening and a partial rescue of WT LRRK2-induced shortening, compared to control shRNA. Bars represent mean (± SEM) length of axons expressed as a percent of GFP alone (control/LV-sh-control) from >60 GFP-positive neurons from at least two independent experiments/cultures. ***P<0.001 comparing LV-sh-ArfGAP1 with LV-sh-control for G2019S LRRK2 by one-way ANOVA with Newman-Keuls post-hoc analysis. *ns*, non-significant.

In yeast, deletion of the *GCS1* gene suppresses human LRRK2-induced toxicity [27]. To determine the effects of modulating ArfGAP1 expression on LRRK2-induced toxicity in primary cortical neurons, we assessed the impact of ArfGAP1 gene silencing on LRRK2-induced neurite shortening. Cortical neurons were first infected with lentiviral vectors expressing shRNAs (sh-control and sh-ArfGAP1 #2, refer to Figure 5) and subsequently co-transfected with FLAG-LRRK2 and GFP constructs at a 10∶1 molar ratio (Figure 9A). The length of GFP-positive axonal processes was determined for each condition. Silencing of endogenous ArfGAP1 expression alone fails to influence axon length compared to a non-silencing shRNA control (Figure 9B). The overexpression of G2019S LRRK2 reduces axonal length by ∼30% and remarkably the shRNA-mediated silencing of ArfGAP1 completely rescues this toxic effect (Figure 9B). WT LRRK2 reduces axonal length by ∼15% in this assay and additional silencing of ArfGAP1 leads to a partial rescue of neurite shortening (Figure 9B). The protective effect of ArfGAP1 silencing against G2019S LRRK2-induced neurite shortening could be replicated using an independent shRNA sequence targeting ArfGAP1 (sh-ArfGAP1 #1, refer to Figure 5) thus confirming the specificity of this protective effect (Figure S7). Collectively, these data demonstrate that silencing of ArfGAP1 expression robustly protects against neuronal toxicity induced by G2019S LRRK2 expression.

### LRRK2 and ArfGAP1 synergistically promote neurite shortening

**Figure 10:**
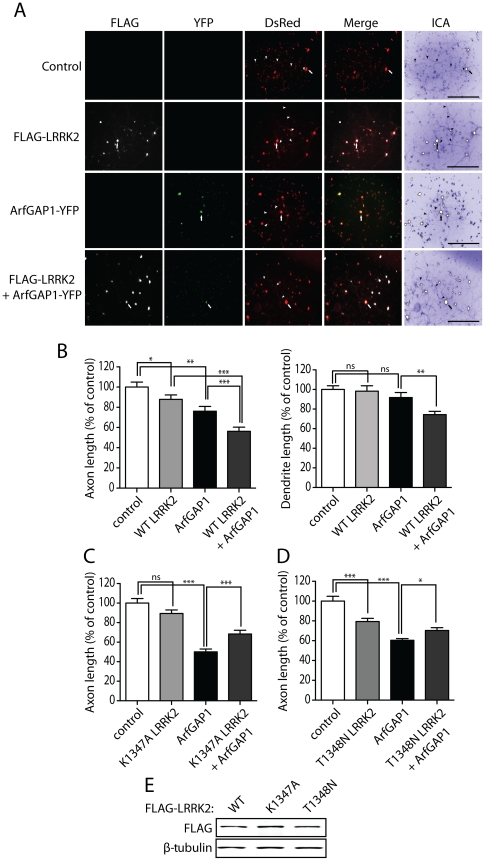
Synergistic effects of LRRK2 and ArfGAP1 overexpression on neurite shortening. (A) Primary cortical neurons were transfected with FLAG-LRRK2 WT, ArfGAP1-YFP and DsRed constructs at a molar ratio of 10∶10∶1 at DIV 3 and fixed at DIV 6. Fluorescent microscopic images indicate the co-labeling of cortical neurons with combinations of FLAG-LRRK2, ArfGAP1-YFP and DsRed. DsRed images were pseudo-colored with ICA for neurite length measurements. Neuronal soma (*arrows*) and axonal processes (*arrowheads*) are indicated. Scale bars: 400 µm. (B) Analysis of DsRed-positive neurites reveals a modest shortening of axons induced by WT LRRK2 or ArfGAP1 expression alone with no effect on dendrite length. Co-expression of WT LRRK2 and ArfGAP1 induces a marked synergistic shortening of axons and dendrites compared to either condition alone. (C) K1347A LRRK2 expression alone has negligible effects on axon length whereas ArfGAP1 alone induces the shortening of DsRed-positive axonal processes. The co-expression of K1347A LRRK2 and ArfGAP1 fails to further reduce axon length but instead modestly protects against ArfGAP1-induced axon shortening. (D) T1348N LRRK2 and ArfGAP1 alone induce the shortening of DsRed-positive axonal processes, whereas their co-expression fails to further reduce axon length. Instead, T1348N LRRK2 expression modestly protects against ArfGAP1-induced axon shortening. Bars represent mean (± SEM) length of axons or dendrites expressed as a percent of DsRed alone (control) from 60–120 DsRed-positive neurons from at least three independent experiments/cultures. *P<0.05, **P<0.01 and ***P<0.001 compared to control (DsRed alone), or by comparison of ArfGAP1 alone with LRRK2/ArfGAP1 co-expression, by one-way ANOVA with Newman-Keuls post-hoc analysis. *ns*, non-significant. (E) Western blot analysis of FLAG-tagged human LRRK2 variants (WT, K1347A or T1348N), used above, following transient expression in HEK-293T cells confirming equivalent LRRK2 protein levels. β-tubulin indicates equivalent protein loading.

Similar to our yeast LRRK2 model [27], reducing ArfGAP1 expression in neurons protects against LRRK2-induced toxicity. Next, we sought to determine the impact of ArfGAP1 overexpression on LRRK2-induced neurite shortening. Primary cortical neurons were co-transfected with combinations of FLAG-LRRK2, ArfGAP1-YFP and DsRed-Max constructs at a 10∶10∶1 molar ratio to morphologically label transfected neurons (Figure 10A). WT LRRK2 was used in these assays in order to assess more subtle effects resulting from co-expression with ArfGAP1. Overexpression of ArfGAP1 alone reduces the length of DsRed-positive axons by ∼20% and dendrites by ∼10%, relative to control neurons expressing DsRed alone (Figure 10B). The overexpression of WT LRRK2 leads to a ∼10% reduction of axon length with negligible effects on dendrite length (Figure 10B). The co-expression of WT LRRK2 and ArfGAP1 in cortical neurons reduces the length of axons (∼45%) and dendrites (∼30%) to a greater extent than would be expected for an additive effect of these proteins (i.e. ∼30% for axons and ∼10% for dendrites) (Figure 10B). Since ArfGAP1 promotes the GTP hydrolysis activity of LRRK2 we reasoned that the synergistic effect on neurite shortening may result from the increased GTPase activity of LRRK2. Accordingly, we assessed the effects of co-expressing the GDP/GTP binding-deficient variant, K1347A LRRK2, together with ArfGAP1 on neurite shortening in cortical neurons. The expression of K1347A LRRK2 alone has a negligible effect on axonal length whereas ArfGAP1 alone markedly reduces axonal length (Figure 10C). The co-expression of these proteins fails to produce a synergistic shortening of axonal processes but instead K1347A LRRK2 modestly protects against ArfGAP1-induced neurite shortening (Figure 10C). A second GTPase-inactive variant, T1348N LRRK2, also fails to produce synergistic effects with ArfGAP1 but similarly partly protects against ArfGAP1-induced neurite shortening (Figure 10D). The modest protective effects of GDP/GTP binding-deficient LRRK2 mutants could potentially result from dominant-negative effects of these variants on endogenous LRRK2. Taken together, these data reveal a synergistic effect of LRRK2 and ArfGAP1 expression on neurite shortening which is dependent, at least in part, on LRRK2 GTPase activity.

### Silencing of LRRK2 expression attenuates ArfGAP1-induced neurite shortening

**Figure 11:**
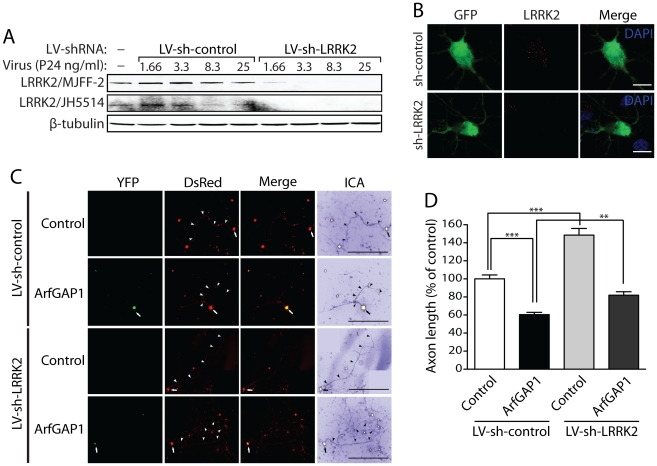
Silencing of LRRK2 expression rescues ArfGAP1-induced neurite shortening. (A) Validation of short hairpin RNA (shRNA)-mediated silencing of endogenous LRRK2 expression in rat primary cortical neuronal cultures. Lentiviral vectors expressing non-silencing shRNA (LV-sh-control) or LRRK2-specific shRNA (LV-sh-LRRK2) were used to infect primary neurons at increasing viral doses ranging from 1.66 to 25 ng of P24 antigen per ml of media. Western blot analysis of neuronal extracts reveals the viral dose-dependent knockdown of LRRK2 with LRRK2-specific shRNAs but not control shRNA using two independent rabbit LRRK2 antibodies (MJFF-2/c41-2 or JH5514). β-tubulin indicates equivalent protein loading. (B) Silencing of endogenous LRRK2 expression in GFP-labeled cortical neurons with lentiviral-shRNA vectors revealed by confocal fluorescence microscopy with a rabbit anti-LRRK2 antibody (JH5514). (C) Primary cortical neurons were infected with lentiviral vectors expressing shRNAs (non-silencing control or LRRK2-specific) at DIV 2, subsequently transfected with ArfGAP1-YFP and DsRed constructs at a 10∶1 molar ratio at DIV 3, and fixed at DIV 6. Fluorescent microscopic images reveal the co-labeling of cortical neurons with ArfGAP1-YFP and DsRed. DsRed images were pseudo-colored with ICA for neurite length measurements. Neuronal soma (*arrows*) and axonal processes (*arrowheads*) are indicated. Scale bars: 400 µm. (D) Analysis of DsRed-positive axonal processes reveals a robust shortening of axons induced by ArfGAP1 expression and increased axon length induced by silencing of LRRK2 with LV-sh-LRRK2 vector, compared to DsRed alone (control). Silencing of LRRK2 with LV-sh-LRRK2 produces a partial rescue of ArfGAP1-induced axon shortening compared to LV-sh-control. Bars represent mean (± SEM) length of axons expressed as a percent of DsRed alone (control/LV-sh-control) from >85 DsRed-positive neurons from two independent experiments/cultures. **P<0.01 and ***P<0.001 compared to control (LV-sh-control/DsRed alone), or by comparison of ArfGAP1/LV-sh-control with ArfGAP1/LV-sh-LRRK2, by one-way ANOVA with Newman-Keuls post-hoc analysis. *ns*, non-significant.

ArfGAP1 overexpression unexpectedly promotes neurite shortening in cortical neurons similar to the effects of G2019S LRRK2 (refer to Figure 10). Furthermore, the synergistic effects of LRRK2/ArfGAP1 on neurite shortening are prevented by impairing the GDP/GTP binding activity of LRRK2 suggesting a role for active LRRK2 in this process. Since ArfGAP1 additionally serves as a robust substrate of LRRK2-mediated phosphorylation (refer to Figure 7), we elected to determine whether LRRK2 expression is reciprocally required for the neurotoxic effects of ArfGAP1. To silence LRRK2 expression, we first developed a lentiviral shRNA vector targeting rodent LRRK2. Rat primary cortical neurons were infected with lentiviral-shRNAs and endogenous LRRK2 expression was monitored by Western blot analysis of neuronal extracts or by confocal microscopy using two well-characterized LRRK2-specific antibodies (MJFF-2/c41-2 and JH5514 [44], [50], [51], [52]). A LRRK2-specific shRNA could induce the viral dose-dependent knockdown of endogenous LRRK2 levels compared to a non-silencing control shRNA in cortical neurons (Figure 11A). We also demonstrate a loss of LRRK2-positive fluorescent signal in GFP-labeled cortical neurons following lentiviral-shRNA-mediated silencing of endogenous LRRK2 compared to a control shRNA (Figure 11B). To determine the effects of modulating LRRK2 expression on ArfGAP1-induced toxicity in primary cortical neurons, we assessed the impact of LRRK2 gene silencing on ArfGAP1-induced neurite shortening. Cortical neurons were first infected with lentiviral vectors expressing shRNAs (LV-sh-control and LV-sh-LRRK2) and subsequently co-transfected with ArfGAP1-YFP and DsRed constructs at a 10∶1 molar ratio (Figure 11C). The length of DsRed-positive axonal processes was determined for each condition. Silencing of endogenous LRRK2 expression alone markedly increases axonal length compared to a non-silencing control shRNA (Figure 11D), as previously reported [36], [37]. The overexpression of ArfGAP1 alone reduces axonal length and the shRNA-mediated silencing of LRRK2 attenuates this toxic effect (Figure 11D). Collectively, these data demonstrate that neuronal toxicity induced by ArfGAP1 expression is dependent, at least in part, on endogenous LRRK2 expression.

ArfGAP1 expression is required for G2019S LRRK2-induced neurite shortening (refer to Figure 9). To determine whether ArfGAP1 expression also influences the increased neurite length induced by silencing of LRRK2 expression, we examined the impact of co-silencing LRRK2 and ArfGAP1 on neurite length. Cortical neurons were co-infected with lentiviral vectors expressing shRNAs (LV-sh-control, LV-sh-LRRK2 or LV-sh-ArfGAP1 #2), subsequently transfected with a GFP construct to morphologically label individual neurons, and the length of GFP-positive axonal processes were determined (Figure S8). Silencing of LRRK2 expression dramatically increases neurite length and the co-silencing of ArfGAP1 does not influence this LRRK2-dependent neurite phenotype (Figure S8). Therefore, the increased neurite length induced by silencing of endogenous LRRK2 expression occurs independently of ArfGAP1 expression.

## Discussion

Here, we demonstrate a novel functional interaction between LRRK2 and the GTPase-activating protein, ArfGAP1. Both proteins biochemically interact *in vitro*, in mammalian cells and *in vivo* in brain. LRRK2 and ArfGAP1 co-localize within the cytoplasm and at the membrane surface of the Golgi complex and Golgi-derived vesicles. The overexpression of ArfGAP1 or LRRK2 commonly promotes the fragmentation and dispersal of the Golgi complex, and for LRRK2 this effect is more pronounced for the familial G2019S mutant compared to the WT protein. The interaction of LRRK2 and ArfGAP1 occurs at least within the cytoplasm of mammalian cells and may not critically require Golgi membranes. Familial PD mutations in LRRK2 tend to enhance the interaction with ArfGAP1 whereas functional mutations influencing LRRK2 GTPase activity also modulate the interaction with ArfGAP1. ArfGAP1 expression does not influence LRRK2 GTP binding but instead markedly enhances the GTP hydrolysis activity of LRRK2 consistent with its known function as a GAP. Unexpectedly, ArfGAP1 enhances the kinase activity of LRRK2 suggesting a potential requirement of GTP hydrolysis for kinase activation. Conversely, LRRK2 robustly and directly phosphorylates ArfGAP1 suggesting the potential for a reciprocal regulation of its GAP activity. Finally, silencing of ArfGAP1 expression protects against neurite shortening induced by G2019S LRRK2 expression in cortical neurons, whereas the co-expression of LRRK2 and ArfGAP1 promotes neurite shortening in a synergistic manner dependent upon LRRK2 GTPase activity. In a reciprocal manner, endogenous LRRK2 expression is required in part for neurite shortening induced by ArfGAP1 overexpression whereas endogenous ArfGAP1 is not required for increased neurite length induced by LRRK2 silencing. Collectively, this study reveals a novel functional role for ArfGAP1 in regulating the GTPase activity and neuronal toxicity of LRRK2. Modulation of ArfGAP1 activity could potentially provide a promising strategy for attenuating LRRK2-induced neurodegeneration in PD.

The functional interaction of LRRK2 with ArfGAP1 is conserved from yeast to mammals. Our prior studies revealed that deletion of the *GCS1* gene in yeast suppressed toxicity and reversed the vesicular trafficking defect induced by the expression of human LRRK2 [27]. However, the mechanisms underlying these effects remain unclear. In the present study, we translate these observations to mammalian cells and demonstrate that ArfGAP1, the mammalian ortholog of yeast *GCS1*, can functionally modulate LRRK2 activity and toxicity. ArfGAP1 is known to act as a GTPase-activating protein for the small GTPase Arf1 where it participates in the recruitment of coat proteins to the surface of Golgi membranes [39], [42], [43]. COPI (coat protein I), COPII or clathrin-AP-2 complexes are well-characterized coat proteins which act to initiate vesicular transport by coupling vesicle formation with cargo sorting [53]. ArfGAP1 is a component of the COPI complex where it serves to regulate Arf1 through GAP activity-dependent inactivation [39], and also serves as a coat protein that acts as an effector of Arf1 [40], [54], [55], [56], [57]. Recently, ArfGAP1 has also been shown to play a similar role in endocytosis regulated by the coat protein AP-2 [58]. In the present study we identify ArfGAP1 as a novel interacting protein of LRRK2. ArfGAP1 unexpectedly interacts with the N-terminal region of LRRK2, a largely uncharacterized region of this protein containing LRRK2-specific, armadillo and ankyrin repeats [1], [59]. The non-catalytic C-terminal region of ArfGAP1 most likely mediates the interaction with LRRK2, a region that is known to mediate protein-protein interactions [57]. The strength of the interaction between ArfGAP1 and LRRK2 is modulated by the GTPase activity of LRRK2 with a preference for the GTP-bound protein. However, impairing LRRK2 GDP/GTP binding through the introduction of the P-loop T1348N mutation, or increasing the GDP-bound state via the GTPase-hyperactive mutation R1398L, reduces but does not prevent the interaction with ArfGAP1 suggesting that factors other than GTP hydrolysis activity or GDP/GTP binding status may additionally regulate this interaction. The observation that the familial PD mutants, R1441C and Y1699C, increase the interaction of LRRK2 with ArfGAP1 may reflect the impaired GTP hydrolysis and increased GTP binding exhibited by these mutations [20], [27]. We demonstrate that ArfGAP1 enhances the GTP hydrolysis activity of LRRK2 by ∼2.5-fold *in vitro*, an effect which is similar in magnitude to the effect of ArfGAP1 on Arf1 GTPase activity. ArfGAP1 does not influence the steady-state levels of GTP-bound LRRK2 variants. Therefore, ArfGAP1 represents a novel GAP protein for regulating the GTPase activity of LRRK2.

The regulation of LRRK2 activity by protein effectors or extrinsic signals is poorly understood. Until now, only a single protein, ARHGEF7, has been suggested to regulate the GTPase domain of LRRK2 by potentially acting as a GEF to promote the exchange of GDP for GTP [26]. The intrinsic regulation of LRRK2 activity is also unclear. It has previously been proposed that the irreversible binding of non-hydrolyzable GTP analogs to the GTPase domain of LRRK2 may promote its kinase activity [19], [20], whereas substantial evidence clearly demonstrates that an intact functional GTPase domain is critically required for kinase activity [19], [20], [60], [61]. Recent compelling data has shown that LRRK2 kinase activity is dependent upon the capacity for GTP binding but is independent of GTP binding *per se* [21]. This has led to the suggestion that interaction with an unknown guanine nucleotide-binding protein may instead regulate LRRK2 kinase activity in a GTP-dependent manner [21]. Therefore, based on the effects of synthetic GTPase mutations (i.e. T1343G/R1398Q or R1398L) that exhibit enhanced GTPase activity yet reduced kinase activity [16], [27], [60], we might anticipate that enhancing GTP hydrolysis activity via ArfGAP1 would similarly reduce the kinase activity of LRRK2. Unexpectedly, we observe that ArfGAP1 enhances the kinase activity of LRRK2. Since ArfGAP1 does not influence the steady-state levels of GTP-bound LRRK2 in cells, our data may suggest that GTP hydrolysis *per se* could serve to regulate kinase activity through an unknown mechanism. Such a mechanism might involve a GTP hydrolysis-dependent alteration in protein conformation which exposes the kinase activation loop or modulates the interaction with a guanine nucleotide-dependent binding protein. Alternatively, it is possible that ArfGAP1 interacts directly with LRRK2 *in vitro* to stabilize the kinase-active dimeric LRRK2 conformation and/or alter the kinase domain thereby prolonging an activated state [62]. Future studies will aim to clarify the mechanism by which ArfGAP1 regulates the kinase activity of LRRK2 both *in vitro* and in mammalian cells.

In a reciprocal manner, we also demonstrate that ArfGAP1 is a robust and direct substrate of LRRK2-mediated phosphorylation *in vitro*. While our data is suggestive of ArfGAP1 being a physiological substrate of LRRK2 kinase activity, further work is required to confirm LRRK2-dependent phosphorylation of ArfGAP1 *in vivo*. The role of LRRK2-mediated ArfGAP1 phosphorylation is unclear. It could serve to reciprocally regulate GAP activity through a positive or negative feedback mechanism, or may serve to regulate the interaction of ArfGAP1 with LRRK2 or other protein factors. ArfGAP1 overexpression in cells results in excessive inactivation of Arf1 and Golgi fragmentation [40], and synergistically promotes neurite shortening when co-expressed with LRRK2, suggesting that LRRK2-mediated phosphorylation does not lead to an inactivation of ArfGAP1 but rather may enhance its activity. Supporting such a mechanism, neurite shortening induced by ArfGAP1 overexpression is dependent, at least in part, upon endogenous LRRK2 expression. It has not yet been possible to confirm whether phosphorylation of ArfGAP1 promotes or inhibits its GAP activity towards LRRK2 or Arf1 since obtaining sufficient quantities of recombinant phosphorylated ArfGAP1 for such GTPase assays has proven difficult. In future studies, the detailed mapping of ArfGAP1 phosphorylation sites and the development of phospho-mimic and phospho-deficient forms will help to determine the impact of LRRK2-dependent phosphorylation on ArfGAP1 activity and neurotoxicity.

The functional interaction of LRRK2 with ArfGAP1 may support a role for LRRK2 in regulating COPI-dependent trafficking of Golgi-derived vesicles as well as AP-2-dependent endocytosis [57], [58]. LRRK2 is localized to a number of vesicular and membranous intracellular structures in mammalian neurons, including lysosomes, clathrin-coated endosomes, Golgi-derived vesicles, microtubule-associated vesicles, multivesicular bodies, the Golgi complex, endoplasmic reticulum and the mitochondrial outer membrane [44], [45], [46]. LRRK2 does not contain obvious transmembrane domains and most likely interacts with proteins or protein complexes at the surface of membranes [63]. In a yeast model, LRRK2 induces defects in the trafficking of endosomes from the plasma membrane to the vacuole most likely due to the abnormal accumulation of autophagic vacuoles in and around this structure [27]. Disruption of the ArfGAP1 ortholog *GCS1* rescued the endosomal trafficking defect and toxicity induced by human LRRK2 in yeast [27]. The expression of human LRRK2 is also known to cause derangements in a number of vesicular trafficking pathways in cells and *in vivo*. In the brains of transgenic mice, the expression of human R1441C and G2019S LRRK2 leads to the abnormal accumulation of autophagic vacuoles [35]. Furthermore, G2019S LRRK2 inducible transgenic mice exhibit neuronal Golgi fragmentation [64], an effect similarly observed in the present study following overexpression of WT or G2019S LRRK2 in primary cortical neurons. In cultured neurons, G2019S LRRK2 expression leads to the accumulation of spheroid axonal inclusions composed of swollen lysosomes, multivesicular bodies, and distended vacuolated mitochondria [36]. The expression of R1441G LRRK2 causes the accumulation of autophagic vacuoles and multivesicular bodies in cultured neurons [46]. Therefore, it is clear that human LRRK2 expression is capable of regulating vesicular trafficking pathways in cells and neurons.

It is not yet clear whether ArfGAP1 regulates the effects of LRRK2 on vesicular trafficking. While ArfGAP1 predominantly localizes to Golgi membranes as part of the COPI complex where it also serves to regulate Arf1 activity [57], it is also found in the cytoplasm of mammalian cells and associates with synaptosomes in mouse brain. The interaction of ArfGAP1with LRRK2 can occur within the cytoplasm of cells, and also potentially at vesicle membranes, where it is likely to have broad implications. In the brain, LRRK2 and ArfGAP1 are detected together in synaptosomal membrane and cytosolic fractions in addition to heavy membrane fractions. The observation that ArfGAP1 can regulate vesicle generation through regulating at least two of the three major vesicle coat proteins (i.e. COPI and clathrin-AP-2) implies that it may influence the generation, trafficking and sorting of diverse vesicular structures [57], [58]. LRRK2 has been reported to interact with the endosomal protein Rab5b to regulate synaptic vesicle endocytosis in neurons [65]. Our recent studies have further shown that LRRK2 can regulate synaptic vesicle exocytosis in addition to endocytosis in neurons [27]. Another recent study demonstrates a role for LRRK2 in synaptic vesicle trafficking and distribution where it may regulate the storage and mobilization of synaptic vesicles within the recycling pool [66]. LRRK2 is present within the synaptosomal compartment of neurons where it has been shown to interact with several pre-synaptic proteins involved in vesicular endocytosis and recycling including AP-2 complex subunits α2 and β1, AP-1 complex subunits α1 and β1, clathrin coat assembly protein AP180, clathrin heavy chain 1 and dynamin-1 [66]. The functional relationship of LRRK2 with these vesicular proteins is unclear but could potentially be related to a proposed function of LRRK2 in regulating actin cytoskeleton dynamics [38], [67]. In future studies, it will be important to determine whether ArfGAP1 plays a role in LRRK2-dependent vesicular trafficking through the regulation of AP-2-mediated endocytosis at the pre-synapse either through direct phosphorylation by LRRK2 or by enhancing LRRK2 GTPase activity. A role for ArfGAP1 in synaptic vesicle endocytosis has not yet been demonstrated but is plausible given its association with synaptosomal membranes.

We demonstrate that neurite shortening induced by G2019S LRRK2 in cortical neurons is regulated by ArfGAP1 expression. Similar to our recent yeast model of LRRK2-dependent toxicity [27], we demonstrate that silencing of ArfGAP1 expression protects against LRRK2-induced neurite shortening, whereas co-expression of ArfGAP1 and LRRK2 promotes this phenotype through a GTPase-dependent mechanism. The molecular basis for these effects is not clear at present. Neurite shortening induced by LRRK2 appears to be more prominent for the G2019S and I2020T kinase domain mutations, whereas the R1441G mutation has only modest effects, with minimal effects for WT LRRK2 [36]. In this study, we demonstrate a small effect of WT and T1348N LRRK2, but not K1347A LRRK2, on axonal length which potentially relates to a GTPase activity-independent effect of LRRK2 in this assay, whereas G2019S LRRK2 induces robust neurite shortening. At least for the G2019S mutation, LRRK2-induced neurite shortening is dependent on kinase activity and requires activation of autophagy [36], [37]. Although the contribution of GTPase activity has not been directly assessed, our data suggest a requirement of ArfGAP1 for LRRK2-induced neurite shortening. Conceivably, this could be due to a direct physiological effect of ArfGAP1 on enhancing the GTP hydrolysis and/or kinase activity of LRRK2 in neurons, as supported by the effects of LRRK2/ArfGAP1 co-expression on neurite length, or may relate to an indirect downstream effect of ArfGAP1 on regulating vesicular trafficking pathways potentially including autophagy [57], [58]. The development of selective inhibitors of ArfGAP1 activity is warranted to determine whether ArfGAP1 inhibition provides a promising strategy for attenuating the pathogenic effects of familial LRRK2 mutants in neurons. Furthermore, it will be important to confirm the contribution of ArfGAP1 to G2019S LRRK2-induced dopaminergic neurodegeneration *in vivo* in available rodent models. We also find that ArfGAP1 expression alone induces robust neurite shortening of cortical neurons that can be attenuated, at least in part, by silencing of endogenous LRRK2 expression. This might suggest that LRRK2-mediated phosphorylation is required for the toxic effects of ArfGAP1. The future identification of ArfGAP1 phosphorylation sites will be required to test this idea. LRRK2 and ArfGAP1 may therefore operate together in a common pathway where they interplay to regulate Golgi integrity and neurite morphology.

Collectively, we demonstrate a novel functional interaction between LRRK2 and ArfGAP1 which serves to regulate LRRK2 GTPase activity and neuronal phenotypes. We identify ArfGAP1 as a novel GAP protein for regulating LRRK2 GTPase activity, whereas ArfGAP1 also represents a new substrate of LRRK2-mediated phosphorylation. ArfGAP1 may represent a promising target for interfering with LRRK2-dependent neurodegeneration in familial and sporadic PD.

## Materials and Methods

### Animals

Mice and rats were housed and treated in strict accordance with the Swiss legislation (Canton de Vaud, Animal Authorization No. 2293) and the European Community Council directive (2010/63/EU) for the care and use of laboratory animals. Animals were maintained in a pathogen-free barrier facility and exposed to a 12 h light/dark cycle with food and water provided *ad libitum*. Pregnant female Sprague-Dawley rats were obtained from Charles River Laboratories (L'Arbresle Cedex, France) and resulting P0 rats were used for preparation of post-natal primary neuronal cultures.

### Expression plasmids, proteins, and antibodies

Mammalian expression plasmids containing FLAG-tagged full-length human LRRK2 (WT, R1441C, Y1699C and G2019S) and FLAG-tagged human LRRK2 deletion mutants were kindly provided by Dr. Christopher Ross (Johns Hopkins University, Baltimore, USA) [30]. Functional GTPase missense mutations (K1347A, T1348N and R1398L) were introduced into FLAG-tagged WT LRRK2 by site-directed mutagenesis using the Stratagene QuickChange II XL kit (Agilent Technologies, La Jolla, CA, USA) and verified by DNA sequencing. C-terminal YFP-tagged rat ArfGAP1 (WT and Δ64N) plasmids were kindly provided by Dr. Jennifer Lippincott-Schwartz (National Institutes of Health, Bethesda, USA) [40]. A GFP-tagged full-length human LRRK2 plasmid was kindly provided by Dr. Mark Cookson (National Institutes of Health, Bethesda, USA) and GFP-tagged human tau was kindly provided by Dr. Leonard Petrucelli (Mayo Clinic, Jacksonville, USA). A plasmid containing C-terminal CFP-tagged human Arf1 was obtained from Addgene (plasmid #11381, [68]). A pRK5 plasmid containing myc-tagged human α-synuclein was kindly provided by Dr. Ted Dawson (Johns Hopkins University, Baltimore, USA). A FLAG-tagged human parkin plasmid was described previously [69]. A pEGFP-N1 plasmid was obtained from Clontech (Mountain View, CA, USA) and a pDsRed-Max-N1 plasmid was obtained from Addgene (plasmid #21718, [70]). Short hairpin RNA (shRNA) sequences in lentiviral plasmid pLKO.1 targeting rat ArfGAP1 (sh-ArfGAP1 #1, TRCN0000047321; sh-ArfGAP1 #2, TRCN0000100750) or rat LRRK2 (sh-LRRK2, TRC0000021461) were obtained from Thermo Fisher Scientific (Open Biosystems, Huntsville, AL, USA). A non-silencing control shRNA sequence in lentiviral plasmid pLKO.1 was obtained from Addgene (plasmid #1864, [71]). Recombinant GST-tagged human LRRK2 protein (residues 970–2527) and LRRKtide peptide (RLGRDKYKTLRQIRQ) were obtained from Invitrogen (Carlsbad, CA, USA). GST-tagged full-length human ArfGAP1 protein was obtained from Novus Biologicals (Littleton, CO, USA). The following antibodies were employed: mouse monoclonal anti-FLAG-(M2), anti-FLAG-(M2)-peroxidase, anti-TH (clone TH-2), anti-MAP2 (clone HM-2) and anti-β-tubulin (clone TUB 2.1), and rabbit polyclonal anti-MAP2 and reagent grade IgG from rabbit serum (Sigma-Aldrich, Buchs, Switzerland); mouse monoclonal anti-GFP (clones 7.1 and 13.1), anti-c-myc (clone 9E10) and anti-c-myc-peroxidase (Roche Applied Science, Basel, Switzerland); rabbit polyclonal anti-TH (Novus Biologicals); rabbit monoclonal anti-LRRK2 (clone MJFF2/c41-2; Epitomics Inc., Burlingame, CA, USA); rabbit polyclonal anti-LRRK2 (JH5514) raised to residues 2500–2515 [44], [51]; rabbit polyclonal anti-ArfGAP1 raised to recombinant full-length human ArfGAP1 protein (Proteintech Group Inc., Chicago, IL, USA); rabbit polyclonal anti-ArfGAP1 raised to recombinant rat ArfGAP1 protein (residues 1–257) was generously provided by Dr. Dan Cassel (Technion-Israel Institute of Technology, Haifa, Israel) [39], [47]; rabbit polyclonal anti-Giantin (ab24586; Abcam, Cambridge, UK); rabbit monoclonal anti-PDI (clone C81H6; Cell Signaling Technology, Danvers, MA, USA); mouse monoclonal anti-TIM23 (clone 32), anti-GM130 (clone 35) and anti-α-synuclein (clone 42) (BD Biosciences, Allschwil, Switzerland); mouse monoclonal anti-synaptophysin 1 (Synaptic Systems, Göttingen, Germany); peroxidase-coupled anti-mouse and anti-rabbit IgG, light chain-specific secondary antibodies (Jackson ImmunoResearch, Inc., West Grove, PA, USA); anti-rabbit IgG and anti-mouse IgG coupled to AlexaFluor-488, -546 and -633 (Invitrogen).

### Cell culture and transient transfection

HEK-293FT cells were maintained in Dulbecco's modified Eagle's media supplemented with 10% foetal bovine serum and 1× penicillin/streptomycin at 37°C in a 5% CO_2_ atmosphere. For transient transfection, cells were transfected with plasmid DNAs using FuGENE HD reagent (Roche Applied Science) according to manufacturer's recommendations. Cells were routinely harvested at 48–72 h post-transfection for biochemical assays.

### Lentivirus production

Lentiviral vectors were produced in HEK-293T cells using a third generation packaging system by calcium phosphate transfection with the following plasmids: pCMV-Δ8.92 (13 µg), pRSV-Rev (3.75 µg), pMD2.G (3 µg) and pLKO.1 vector containing shRNA sequence (13 µg) [72]. After 72 h the medium was collected and centrifuged in a SW32Ti ultracentrifuge rotor at 19,000 rpm for 90 min at 4°C. The pellet was resuspended in 3 ml of buffer containing 1× PBS pH 7.4 and 0.5% BSA for a 50× concentrated virus stock. Viral titer was determined using the HIV-1 p24 antigen ELISA kit (Zeptometrix Corp., Buffalo, USA). A p24 of 1.66 to 50 ng/ml was used for infecting primary cortical neurons at a density of 400,000 cells in 3 ml media (per 35 mm dish).

### Co-immunoprecipitation assay and Western blot analysis

For co-immunoprecipitation (IP) assays, HEK-293FT cells were transiently transfected with each plasmid in 10 cm dishes. After 48 h, confluent cells were harvested in 1 ml of IP buffer (1× phosphate-buffered saline [PBS] pH 7.4, 1% Triton X-100, 1× phosphatase inhibitor cocktail 1 and 2 [Sigma-Aldrich], 1× Complete Mini protease inhibitor cocktail [Roche Applied Sciences]). Cell lysates were rotated at 4°C for 1 h and soluble fractions were obtained by centrifugation at 17,500 *g* for 15 min at 4°C. Soluble fractions were combined with 50 µl Protein G-Dynabeads (Invitrogen) pre-incubated with mouse anti-FLAG (5 µg; Sigma-Aldrich), anti-GFP (1 µg; Roche Applied Sciences) or anti-myc (5 µg; Roche Applied Sciences) antibodies followed by overnight incubation at 4°C. Dynabead complexes were sequentially washed once with IP buffer supplemented with 500 mM NaCl, twice with IP buffer and three times with PBS. Immunoprecipitates were eluted by heating at 70°C for 10 min in 2× Laemmli sample buffer (Bio-Rad AG, Reinach, Switzerland) with 5% 2-mercaptoethanol. IPs and inputs (1% total lysate) were resolved by SDS-PAGE, transferred to Protran nitrocellulose (0.2 µm; Perkin Elmer, Schwerzenbach, Switzerland), and subjected to Western blot analysis with appropriate primary and secondary antibodies. Proteins were visualized by enhanced chemiluminescence (ECL; GE Healthcare, Glattbrugg, Switzerland) on a FujiFilm LAS-4000 Luminescent Image Analysis system. Quantitation of protein levels by densitometry was conducted on acquired images using LabImage 1D software (Kapelan Bio-Imaging Solutions, Leipzig, Germany).

For *in vitro* pull-down assays with recombinant proteins, HEK-293T cell extracts expressing FLAG-LRRK2 or FLAG-parkin, or mock transfected, were subjected to IP with anti-FLAG antibody (5 µg) and Protein G-Dynabeads (Invitrogen) overnight at 4°C and washed stringently five times with IP buffer supplemented with 500 mM NaCl, and once with PBS. FLAG IP-Dynabead complexes were combined with recombinant GST-ArfGAP1 (500 ng) in 1× PBS and incubated overnight at 4°C. Dynabead complexes were washed three times with IP buffer and subjected to Western blot analysis with anti-ArfGAP1 and anti-FLAG antibodies.

For *in vivo* co-IP, protein extracts were prepared from the cerebral cortex of adult wild-type and LRRK2 knockout mice (with targeted deletion of exon 41 of the *LRRK2* gene [73]; generously provided by Drs. Giorgio Rovelli and Derya Shimshek, Novartis Pharma AG, Basel, Switzerland) by homogenization in TNE buffer (10 mM Tris-HCL pH 7.4, 150 mM NaCl, 5 mM EDTA, 0.5% NP-40, 1× phosphatase inhibitor cocktail 1 and 2 [Sigma-Aldrich], 1× Complete Mini protease inhibitor cocktail [Roche Applied Sciences]). Protein concentration was determined by BCA assay (Pierce Biotechnology, Rockford, IL, USA). Brain extracts (10 mg protein) were combined with 50 µl Protein G-Dynabeads (Invitrogen) pre-incubated with rabbit anti-LRRK2 (5 µg; MJFF2/c41-2; Epitomics, Inc.), rabbit anti-ArfGAP1 (3 µg; Proteintech Group Inc.) or rabbit IgG (3 µg; Sigma-Aldrich) antibodies followed by overnight incubation at 4°C. Dynabead complexes were sequentially washed twice with TNE buffer and twice with TBS buffer (10 mM Tris-HCL pH 7.4, 150 mM NaCl). Immunoprecipitates were eluted by heating at 70°C for 10 min, resolved by SDS-PAGE and subjected to Western blot analysis.

For western blot analysis of mouse brain tissues, adult C57BL/6J mice were sacrificed and anatomic brain regions were rapidly dissected and frozen on dry ice. Protein extracts were prepared from brain tissues by homogenization in TNE buffer, and clarified by centrifugation at 100,000 *g* for 20 min at 4°C. The detergent-soluble supernatant fraction was quantified by BCA assay (Pierce Biotechnology) and 75–100 µg of protein was resolved by SDS-PAGE and subjected to Western blot analysis with rabbit anti-ArfGAP1 (provided by Dr. D. Cassel), rabbit anti-LRRK2 (clone c41-2; Epitomics, Inc.) and mouse anti-β-tubulin (clone TUB 2.1; Sigma-Aldrich) antibodies.

### Immunocytochemistry and confocal microscopy

For co-localization of LRRK2 and ArfGAP1, HEK-293FT cells or primary cortical cultures transiently expressing FLAG-LRRK2 and ArfGAP1-YFP were fixed in 4% paraformaldehyde (PFA) and processed for immunocytochemistry with mouse anti-FLAG-(M2) antibody, and anti-mouse IgG-AlexaFluor-633 antibody. Endogenous ArfGAP1 was visualized using a rabbit anti-ArfGAP1 antibody (provided by Dr. D. Cassel) and anti-rabbit IgG-AlexaFluor-488 antibody. For localization of endogenous ArfGAP1 to neurons, primary cortical and midbrain cultures were fixed and processed for immunocytochemistry with rabbit anti-ArfGAP1 antibody (provided by Dr. D. Cassel) and either mouse anti-TH antibody (for midbrain cultures) or mouse anti-MAP2 antibody (for cortical cultures), and anti-rabbit-IgG-AlexFluor-488 and anti-mouse IgG-AlexaFluor-633 antibodies. For co-localization of LRRK2 or ArfGAP1 with Golgi markers, FLAG-LRRK2 (anti-FLAG antibody), endogenous LRRK2 (JH5514 antibody, [44]) or endogenous ArfGAP1 (anti-ArfGAP1; provided by Dr. D. Cassel) were combined with either anti-GM130 or anti-Giantin antibodies, and visualized with appropriate anti-IgG-AlexaFluor secondary antibodies. Fluorescent images were acquired using a Zeiss LSM 700 inverted confocal microscope (Carl Zeiss AG, Feldbach, Switzerland) with a Plan-Apochromat 63×/1.40 oil objective in x, y and z planes. Images were subjected to deconvolution using HuygensPro software (Scientific Volume Imaging, Hilversum, Netherlands). Representative images are taken from a single z-plane at a thickness of 0.1 to 1 µm.

### Subcellular fractionation of brain tissue

Subcellular fractionation was conducted as described previously [44], [74] using whole brain tissue from adult C57BL/6J mice. Briefly, mouse brain homogenates were subjected to centrifugation at 800 *g* for 10 min to generate pellet (P1, nuclear/whole cell) and soluble (S1, cytosolic) fractions. S1 fractions were centrifuged at 9,200 *g* for 15 min to produce P2 (heavy and crude synaptosomal membranes) and S2 (soluble cytosolic) fractions. The P2 fraction was solublized and centrifuged at 25,000 *g* for 20 min to produce LP1 (synaptosomal membranes) and LS1 (synaptosomal cytosolic) fractions. The LS1 fraction was further fractionated by ultracentrifugation at 165,000 *g* for 2 h to produce LP2 (synaptic vesicle-enriched) and LS2 (synaptic vesicle cytosolic) fractions. The S2 fraction was subjected to ultracentrifugation at 165,000 *g* for 2 h to produce P3 (light membranes/microsomes) and S3 (soluble cytosolic) fractions. Protein concentrations were determined by BCA assay (Pierce Biotechnology) and equal quantities of each fraction were validated by Western blotting with specific antibodies labeling mitochondria (TIM23; P2 and LP1), endoplasmic reticulum (PDI; P2, LP1 and LP2), Golgi complex (Giantin; P2 and P3), synaptosomes/synaptic vesicles (synaptophysin 1; P2, P3, LP1 and LP2), and synaptosomal/synaptic vesicle cytosolic (α-synuclein; LS1 and LS2) subcellular compartments.

### Subcellular fractionation of HEK-293T cells

HEK-293T cells were transfected with combinations of FLAG-LRRK2 and ArfGAP1-YFP plasmids for 48 h. Confluent cells were harvested in 1× PBS and homogenized by 10 strokes in a 2 ml glass Dounce homogenizer with a polytetrafluoroethylene pestle. Total homogenates (H) were centrifuged at 1,000 *g* for 10 min to produce pellet (P1, nuclei/cell debri) and soluble (S1) fractions. The S1 fraction was centrifuged at 10,000 *g* for 10 min to produce pellet (P2, heavy membranes) and soluble (S2) fractions. The S2 fraction was subjected to ultracentrifugation at 100,000 *g* for 1 h to produce pellet (P3, light membranes) and soluble (S3, cytosolic) fractions. Protein concentrations were determined by BCA assay (Pierce Biotechnology) and equal quantities of each fraction were validated by Western blotting with specific antibodies labeling heavy (P2, anti-TIM23/mitochondria) or light (P3, anti-GM130/Golgi complex) membranes.

For co-immunoprecipitation analysis of FLAG-LRRK2 and ArfGAP1-YFP in subcellular fractions, the membrane-deficient soluble S3 fraction was subjected to IP with anti-FLAG antibody (5 µg; Sigma-Aldrich) coupled to Protein G-Dynabeads (Invitrogen) as described above, and IPs and input lysates were subjected to Western blotting with anti-GFP and anti-FLAG antibodies.

### GTP binding assay

HEK-293T cells transiently expressing FLAG-tagged LRRK2 variants or LRR-Roc (F3, residues 895–1503) were lysed in 1 ml of lysis buffer G (1× PBS pH 7.4, 1% Triton X-100, 1× phosphatase inhibitor cocktail 1 and 2 [Sigma-Aldrich], 1× Complete Mini protease inhibitor cocktail [Roche Applied Sciences]), rotated for 1 h at 4°C, and clarified by centrifugation at 17,500 *g* for 10 min at 4°C. Soluble proteins were incubated with 50 µl γ-aminohexyl-GTP-sepharose bead suspension (Jena Bioscience, Jena, Germany) by rotating for 2 h at 4°C. Beads were washed three times with buffer G and once with PBS alone. For GTP competition assays, incubation was allowed to proceed for 60 min at 4°C, GTP was added to a final concentration of 2–4 mM, and incubation was continued for a further 60 min at 4°C followed by washing. GTP-bound proteins were eluted in 2× Laemmli sample buffer containing 5% 2-mercaptoethanol by heating at 70°C for 10 min. GTP-bound proteins or input lysates (1% total lysate) were resolved by SDS-PAGE and subjected to Western blotting with anti-FLAG and anti-GFP antibodies.

### GTP hydrolysis assay

GTP hydrolysis activity was measured as previously described [27] by monitoring the release of free γ-phosphate (P_i_) from GTP. Briefly, HEK-293FT cells transiently expressing full-length FLAG-LRRK2 variants (WT, T1348N or R1398L), Arf1-CFP or ArfGAP1-YFP (WT or ΔN) in 10 cm dishes were lysed in 1 ml of phosphate-free lysis buffer (10 mM Tris-HCl pH 7.5, 150 mM NaCl, 1% NP-40, 1× Complete Mini protease inhibitor cocktail [Roche Applied Sciences]) and subjected to immunoprecipitation (IP) with anti-FLAG (5 µg) or anti-GFP (1 µg) antibodies pre-incubated with 50 µl Protein G-Dynabeads (Invitrogen) by rotating at 4°C overnight. Similar non-transfected HEK-293FT cell lysates were also subjected to IP with anti-FLAG or anti-GFP antibodies to control for non-specific protein contamination. Dynabeads were stringently washed 3× with lysis buffer and 2× with 0.5 M Tris-HCl pH 7.5, finally resupended in 45 µl of 0.5 M Tris-HCl pH 7.5, and subjected to GTP hydrolysis assays in 96-well plates using the high sensitivity colorimetric GTPase assay kit (Innova Biosciences, Cambridge, UK) as per manufacturer's recommendations. To control for protein contamination, each well received 20 µl of FLAG- and GFP-coupled Dynabeads at a 1∶1 ratio (total 40 µl Dynabead suspension) to a final volume of 100 µl in 0.5 M Tris buffer before addition of 100 µl substrate buffer mix containing 0.5 mM GTP. For Arf1 hydrolysis assays, each well received a total of 40 µl of GFP-coupled Dynabead suspension. In parallel, replicate samples were set up in the absence of GTP to account for non-specific background absorbance. Assay samples were incubated for 2 h at room temperature, Dynabeads were carefully removed to prevent interference with absorbance measurements, and the remaining assay sample was measured for absorbance at 635 nm. Absorbance from samples in the absence of GTP was removed from the equivalent sample containing GTP. The resulting absorbance value was used to calculate P_i_ concentration based on a known P_i_ standard curve included on each 96-well plate. Data were expressed as a percent of P_i_ release due to WT LRRK2 or Arf1 alone. Each anti-FLAG or anti-GFP IP used above (5 µl total) was subjected to Western blotting with anti-FLAG or anti-GFP antibodies to confirm immuno-purification of LRRK2, Arf1 or ArfGAP1 proteins in each experiment. The relative expression levels of each LRRK2 variant was determined by densitometry and used to normalize LRRK2-mediated P_i_ release in each experiment.

### *In vitro* kinase assays

Recombinant commercially purified human WT, R1441C, G2019S and D1994A LRRK2 (Δ1-970) proteins were purchased from Invitrogen for use in some experiments. Recombinant proteins were assessed for equal purity (>95% by Coomassie SDS-PAGE) and protein concentration as determined by BCA assay (Pierce Biotechnology). Recombinant human full-length myc-tagged human LRRK2 protein was isolated from HEK-293FT cells (Invitrogen) transiently transfected with LRRK2-myc plasmids. Cells were collected at 48 h post-transfection and centrifuged at 500 *g* for 5 min. Pelleted cells were re-suspended in lysis buffer (0.5% Triton X-100, 1× Complete protease inhibitor cocktail (Roche Applied Sciences) and 1× PhoStop (Roche Applied Sciences) in 1× PBS pH 7.4 without Ca^2+^ or Mg^2+^) and rotated at 4°C for 1 h. Lysates were clarified by centrifugation at 20,000 *g* for 10 min. Supernatant was incubated with anti-c-myc antibody (clone 9E10, Roche Applied Sciences) pre-incubated with Dynabeads-Protein G (Invitrogen) for 16 h. Supernatants were discarded and beads washed 3× in PBS supplemented with 500 mM NaCl, 3× in PBS and resuspended in kinase buffer (5 mM EGTA and 20 mM β-glycerol phosphate in PBS). Reactions were initiated by addition of activation buffer to final concentrations that includes 0.1 mM [^32^P]-γ-ATP (0.2 µCi/reaction) and 20 mM MgCl_2_ (reactions using ArfGAP1 were conducted with 1 µg of protein) and incubation at 30°C with shaking for 30 min. Reactions were terminated by placing the tubes in ice and removing supernatant to P-81 Whatman paper for scintillation counting of LRRKtide peptide phosphorylation. The remaining beads and reaction buffer were suspended in 2× Laemmli sample buffer. Reactions were then heated at 75°C for 10 min and resolved on Tris-acetate SDS-PAGE gels. Immunoprecipitated proteins were stained using Coomassie G-250 (Bio-Rad) according to the manufacturer's protocol. Whatman P-81 discs were washed 5× in 100 mM phosphoric acid buffer or with additional washes until no radioactivity could be detected in wash buffer. For comparisons in levels of kinase activity against peptide substrates, one-way ANOVA and Newman-Keuls post-hoc test were used to determine significance.

### Primary neuronal cultures

Whole brains were dissected from Sprague-Dawley P0 rats and the cerebral cortices and ventral midbrain (containing the substantia nigra and ventral tegmental area) were stereoscopically isolated and dissociated in media containing papain (20 U/ml; Sigma). The cells were grown in 35 mm dishes on glass coverslips pre-coated with mouse laminin (33 µg/ml; Invitrogen) and poly-*D*-lysine (20 ng/ml; BD Biosciences, Allschwil, Switzerland) in media consisting of Neurobasal (Invitrogen), B27 supplement (2% w/v), L-glutamine (500 µM) and penicillin/streptomycin (100 U/ml). At *days-in-vitro* (DIV) 3, cortical and midbrain cultures were treated with cytosine β-*D*-arabinofuranoside (AraC, 10 µM) to inhibit glial cell division. By immunocytochemical analysis with neuronal (MAP2 and TH) and astrocyte (GFAP) markers, cortical neurons at DIV 6 were shown to consist of MAP2-positive neurons (36.20±2.03% of total cells), MAP2-negative neurons (45.61±2.21%) and GFAP-positive astrocytes (18.19±2.10% of total) based upon random sampling from 10 independent microscopic fields at 10× magnification. Midbrain cultures at DIV 6 consist of TH-positive dopaminergic neurons (6.4±0.9% of total cells), MAP2-positive neurons (51.63±2.10% of total), GFAP-positive astrocytes (28.34±2.36% of total) and other cells (20.02±3.10% of total).

### Neurite length assays

#### Cortical neurite length assay

Primary cortical cultures at DIV 3 were co-transfected with FLAG-LRRK2 and DsRed-Max, GFP or GFP-tau plasmids at a 10∶1 molar ratio (5 µg total DNA per 35 mm dish) using Lipofectamine 2000 reagent (Invitrogen) according to manufacturer's recommendations. At DIV 6, cultures were fixed with 4% paraformaldehyde and processed for immunocytochemistry with mouse anti-FLAG-(M2) antibody (Sigma-Aldrich) and anti-mouse IgG-AlexaFluor-488 or -633 antibodies (Invitrogen). For assays using shRNAs, cultures were infected at DIV 2 with lentiviral vectors expressing shRNAs (non-silencing control or ArfGAP1-specific shRNAs) at a final dose of 3.3 ng of p24 antigen per ml of media, followed by co-transfection as described above. Fluorescent images were acquired using an EVOS inverted fluorescence digital microscope (Advanced Microscopy Group, Bothell, WA, USA) with a 10× objective. DsRed or GFP images were pseudo-colored using ICA1 in NIH Image J software to improve the contrast of neuritic processes, and used for neurite length measurements. The length of DsRed-positive neurites from single (control: DsRed) or double-positive (LRRK2: DsRed/FLAG) cortical neurons were measured using the line tool function of NIH ImageJ software by an investigator blinded to each condition. Only neurons that had extended neurites were measured whereas neurons without processes were excluded from the analysis. DsRed-positive neurites were assigned as axons (longest neurite) or dendrites (all neurites minus the longest neurite). In each experiment, neuronal processes from 50–100 DsRed-positive neurons randomly sampled across five coverslips from at least two independent experiments were measured. For assays using GFP-tau, the length of the longest axonal process following branching was measured as described above from 76–87 individual GFP-tau-positive neurons sampled from two independent cultures.

For co-expression experiments, primary cortical cultures at DIV 3 were transfected with FLAG-LRRK2, ArfGAP1-YFP and DsRed plasmids at a 10∶10∶1 molar ratio (5 µg total DNA per 35 mm dish) using Lipofectamine 2000 reagent (Invitrogen). Cells were fixed at DIV 6 and processed for immunocytochemistry with mouse anti-FLAG-(M2) antibody (Sigma-Aldrich) and anti-mouse IgG-AlexaFluor-633 antibody (Invitrogen). Fluorescent images were acquired and the length of DsRed-positive neurites from single (control: DsRed), double-positive (LRRK2 or ArfGAP1: DsRed/FLAG or DsRed/YFP) or triple-positive (LRRK2+ArfGAP1: DsRed/FLAG/YFP) cortical neurons were measured using NIH ImageJ software as described above.

For LRRK2 silencing experiments, primary cortical neurons were infected at DIV 2 with lentiviral vectors expressing shRNAs (non-silencing control or LRRK2-specific shRNA) at a final dose of 3.3 ng of p24 antigen per ml of media, followed by co-transfection at DIV 3 with ArfGAP1-YFP and DsRed plasmids at a 10∶1 molar ratio. Cells were fixed at DIV 6, fluorescent images were acquired and the length of DsRed-positive neurites from single (shRNA alone: DsRed) or double-positive (ArfGAP1+shRNA: DsRed/YFP) cortical neurons were measured using NIH ImageJ software as described above.

#### Dopaminergic neurite length assay

Primary midbrain cultures at DIV 3 were transfected with FLAG-LRRK2 and GFP plasmids at a 10∶1 molar ratio as described above and fixed at DIV 6. Cells were processed for immunocytochemistry with mouse anti-FLAG-(M2) (Sigma-Aldrich) and rabbit anti-TH (Novus Biologicals) antibodies, and anti-mouse IgG-AlexaFluor-633 and anti-rabbit IgG-AlexaFluor-546 antibodies (Invitrogen). The length of GFP-positive neurites from double-positive (control: GFP/TH) or triple-positive (LRRK2: GFP/TH/FLAG) dopaminergic neurons were measured using NIH Image J software as described above.

### TUNEL labeling

Primary cortical neurons were co-transfected with FLAG-LRRK2 (or empty vector) and GFP plasmids at a 10∶1 molar ratio at DIV 11 and fixed with 4% PFA at DIV 14. TUNEL staining was conducted using the *In Situ* Cell Death Detection Kit (Roche Applied Sciences) containing tetramethylrhodamine (TMR) red-labeled dUTP as per the manufacturer's instructions. Cultures were further subjected to immunocytochemistry with mouse anti-FLAG-(M2) antibody (Sigma-Aldrich) and anti-mouse IgG-AlexaFluor-633 antibody (Invitrogen). Fluorescent microscopic images were acquired of individual single (control: GFP) or double-positive (LRRK2: FLAG/GFP) cortical neurons and the proportion of TUNEL-positive nuclei was scored. In each experiment, the number of TUNEL-positive nuclei from GFP-positive or GFP/FLAG-positive neurons (*n* = 155–174) randomly sampled across three coverslips from three independent cultures were measured. Data represent TUNEL-positive neurons as a percent of total GFP-positive neurons (mean ± SEM) for each condition.

### Golgi fragmentation assay

Primary cortical neurons at DIV 3 were transfected with FLAG-LRRK2 or pcDNA3.1 control plasmid (4.5 µg DNA per 35 mm dish) using Lipofectamine 2000 reagent (Invitrogen). At DIV 6, cultures were fixed with 4% PFA and processed for immunocytochemistry with mouse anti-FLAG-(M2) (Sigma-Aldrich) and rabbit anti-Giantin (Abcam) antibodies, and anti-mouse-IgG-AlexFluor-546 and anti-rabbit-IgG-AlexaFluor-488 antibodies (Invitrogen). Confocal microscopic analysis was conducted on a Zeiss LSM 700 inverted confocal microscope (Carl Zeiss AG, Feldbach, Switzerland) with a Plan-Apochromat 63×/1.40 oil objective in x, y and z planes. Golgi morphology was assessed in individual control or LRRK2-positive (FLAG) cortical neurons using Giantin immunofluorescence, an endogenous transmembrane protein of the cis and medial Golgi complex. Golgi were classified as either normal (tubular network), intermediate (partially fragmented plus tubular network) or fragmented (fully fragmented without tubular network). In each experiment, Golgi morphology was scored from control (*n* = 498), WT LRRK2-positive (*n* = 359) or G2019S LRRK2-positive (*n* = 238) cortical neurons randomly sampled across five coverslips from three independent cultures. Golgi subclasses were expressed as a percent of the total number of Golgi for each condition.

### Statistical analysis

Data were analyzed by two-tailed, unpaired Student's *t*-test for pair-wise comparisons, or by one-way ANOVA with Newman-Keuls post-hoc analysis for comparison of multiple data groups, as indicated. *P*<0.05 was considered significant.

## Supporting Information

Figure S1 Direct interaction of LRRK2 with ArfGAP1. (A) SDS-PAGE analysis of recombinant full-length GST-tagged human ArfGAP1 (140 or 420 ng protein) by staining with coomassie colloidal blue or by Western blot analysis with rabbit anti-ArfGAP1 antibody (Proteintech Group Inc.). GST-ArfGAP1 was also added to HEK-293T cell lysates (20 µg protein) and detected by probing with anti-ArfGAP1 antibody. Equal loading of cell lysates is indicated by staining with Ponceau S. (B) SDS-PAGE analysis of recombinant FLAG-LRRK2 or FLAG-parkin immunoprecipitated (IP) from transfected HEK-293T cells with anti-FLAG antibody and stained with coomassie colloidal blue. IgG heavy chain (HC) is indicated confirming equal loading of IPs. FLAG IP from non-transfected (untr.) cell lysates was used to assess contaminating proteins (*). (C) *In vitro* interaction of recombinant GST-ArfGAP1 with immunopurified FLAG-LRRK2 but not with FLAG-parkin or mock FLAG IP. GST-ArfGAP1 interaction with each FLAG IP is detected with anti-ArfGAP1 antibody. IgG light chain (LC) is also indicated confirming equal loading of IPs. Molecular mass markers are indicated in kilodaltons.
(TIF)

Figure S2 Confirmation of functional LRR-Roc protein fragment of LRRK2. (A) FLAG-tagged human LRR-Roc (fragment F3, residues 895–1503) and full-length WT LRRK2 bound to GTP following pull-down assays with GTP-sepharose from HEK-293T cells. Confirmation of the specificity of LRR-Roc or WT LRRK2 GTP binding is indicated by negligible binding of the GDP/GTP binding-deficient LRRK2 mutant, T1348N. Competition with an excess of free GTP (4 mM) reduces binding of LRR-Roc or WT LRRK2 to GTP-sepharose. (B) Co-immunoprecipitation of FLAG-tagged LRR-Roc or full-length WT LRRK2 with GFP-tagged full-length LRRK2 from HEK-293T cells following IP with anti-GFP antibody. Both LRR-Roc and WT LRRK2 are capable of forming dimers with GFP-LRRK2 suggesting appropriate folding of the LRR-Roc fragment. Molecular mass markers are indicated in kilodaltons.
(TIF)

Figure S3 Co-localization of ArfGAP1 or LRRK2 with the Golgi complex in mammalian cells and neurons. (A) Confocal fluorescence microscopy reveals the co-localization of endogenous ArfGAP1 with the Golgi membrane marker, GM130, in HEK-293T cells. Exogenous ArfGAP1-YFP also co-localizes with Golgi membrane markers (GM130 or Giantin) to varying degrees depending upon the level of overexpression. ArfGAP1-YFP can localize to the intact Golgi complex (*upper panel*), can induce the fragmentation of the Golgi complex with the appearance of ArfGAP1-YFP-positive Golgi-derived vesicles (*middle panel*), or can induce the complete removal of the Golgi complex with ArfGAP1-YFP-positive Golgi-derived vesicles remaining (*lower panel*). (B) Similar co-localization of endogenous ArfGAP1 or exogenous ArfGAP1-YFP with the Golgi membrane marker, GM130, in rat primary cortical neurons (*upper panels*). ArfGAP1-YFP overexpression induces Golgi fragmentation with the appearance of ArfGAP1-YFP-positive Golgi-derived vesicles that are devoid of Golgi markers. Endogenous LRRK2 (JH5514 antibody) or exogenous FLAG-tagged LRRK2 partially co-localizes with the Golgi membrane markers, GM130 and Giantin, respectively, in primary cortical neurons (*lower panels*). Cytofluorograms and co-localization coefficients (Rcoloc) reveal the extent of co-localization between LRRK2 or ArfGAP1 and Golgi marker fluorescence signals. Confocal images are taken from single z-plane at 0.1 µm thickness. Images are representative of at least three independent experiments. Scale bars: 10 µm.
(TIF)

Figure S4 Full-length LRRK2 phosphorylates ArfGAP1. *In vitro* radioactive kinase assay with full length (FL) immunopurified myc-tagged human LRRK2 variants (WT, R1441C, G2019S and kinase-dead D1994A) and recombinant full-length GST-tagged human ArfGAP1. Autoradiographs (^32^P) reveal ArfGAP1 phosphorylation by WT, R1441C and G2019S LRRK2 but not D1994A LRRK2, with ArfGAP1 phosphorylation levels correlating with levels of LRRK2 autophosphorylation (*upper panel*). (*) indicates the LRRK2-dependent phosphorylation of an unknown LRRK2-interacting protein (>75 kDa) in these reactions. Coomassie colloidal blue-stained SDS-PAGE gels reveal equivalent loading and purity of immunoprecipitated full-length myc-LRRK2 variants (∼260 kDa) in each reaction (*middle panel*). Western blot analysis with anti-GST antibody indicates equivalent loading of GST-ArfGAP1 (∼75 kDa) in each reaction (*lower panel*). Molecular mass markers are indicated in kilodaltons.
(TIF)

Figure S5 Effects of G2019S LRRK2 expression on neurite length of midbrain dopaminergic neurons. (A) Rat primary ventral midbrain cultures were transfected with FLAG-LRRK2 G2019S and GFP constructs at a 10∶1 molar ratio at DIV 3 and fixed at DIV 6. Fluorescent microscopic images indicate the co-labeling of FLAG-LRRK2, GFP and tyrosine hydroxylase (TH). GFP/TH merged images were rendered in ICA for neurite length measurements. Neuronal soma (*arrows*) and axonal processes (*arrowheads*) are indicated. Scale bars: 400 µm. (B) G2019S LRRK2 expression has negligible effects on the length of GFP/TH-positive dopaminergic axons but increases the length of dopaminergic dendrites, compared to GFP alone (control). Bars represent the mean (± SEM) length of axons or dendrites in µm from 30–40 GFP/TH-positive dopaminergic neurons from at least two independent experiments. *P<0.05 compared to control (GFP alone) by two-tailed unpaired Student's *t*-test. *ns*, non-significant.
(TIF)

Figure S6 Effects of G2019S LRRK2 expression on axonal length and cell death in cortical neurons. (A) Rat primary cortical neurons were transfected at DIV 3 with FLAG-LRRK2 G2019S and GFP-tau constructs at a DNA molar ratio of 10∶1. Cultures were fixed at DIV 6. Fluorescent microscopic images reveal the co-labeling of cortical neurons with FLAG-LRRK2 and GFP-tau. GFP images were pseudo-colored with ICA to improve the contrast of axonal processes for length measurements. Neuronal soma (*arrows*) and axonal processes (*arrowheads*) are indicated. Scale bars: 400 µm. (B) Analysis of the length of GFP-tau-positive axonal processes reveals a significant shortening of axons due to G2019S LRRK2 expression, compared to GFP-tau alone (control). Bars represent axon length (mean ± SEM) expressed as a percent of control (GFP-tau alone) from 76–87 GFP-tau-positive neurons from at least two independent experiments/cultures. ***P<0.001 compared to control (GFP-tau alone) by two-tailed unpaired Student's *t*-test. (C) Effect of LRRK2 expression on apoptotic cell death of cortical neurons. Primary cortical neurons were transfected with FLAG-LRRK2 variants and GFP at a 10∶1 molar ratio at DIV 11 and fixed at DIV 14. Cultures were subjected to TUNEL staining and immunocytochemistry with anti-FLAG antibody. TUNEL-positive neurons were counted as a percent of total GFP-positive (control) or GFP/FLAG-positive (LRRK2) neurons and expressed as mean ± SEM (*n* = 3 experiments/cultures).
(TIF)

Figure S7 Silencing of ArfGAP1 expression with lentiviral sh-ArfGAP #1 rescues G2019S LRRK2-induced neurite shortening. (A) Silencing of endogenous ArfGAP1 expression in cortical neurons with a lentiviral ArfGAP1-specific shRNA (LV-sh-ArfGAP1 #1) compared to a non-silencing control shRNA (LV-sh-control) revealed by confocal fluorescence microscopy with a rabbit anti-ArfGAP1 antibody. (B) Primary cortical neurons were infected with lentiviral vectors expressing shRNAs (non-silencing control or ArfGAP1-specific) at DIV 2, subsequently transfected with FLAG-LRRK2 G2019S and GFP constructs at a 10∶1 molar ratio at DIV 3, and fixed at DIV 6. Fluorescent microscopic images reveal the co-labeling of cortical neurons with FLAG-LRRK2 and GFP. GFP images were pseudo-colored with ICA for neurite length measurements. Neuronal soma (*arrows*) and axonal processes (*arrowheads*) are indicated. Scale bars: 400 µm. (C) Analysis of GFP-positive axonal processes reveals a robust shortening of axons induced by G2019S LRRK2 expression compared to GFP alone (control/LV-sh-control). Knockdown of ArfGAP1 with lentiviral-shRNA vectors (LV-sh-ArfGAP1 #1) produces a complete rescue of G2019S LRRK2-induced axon shortening compared to control shRNA (LV-sh-control). Bars represent axon length (mean ± SEM) expressed as a percent of GFP alone (control/LV-sh-control) from >60 GFP-positive neurons from at least two independent experiments/cultures. **P<0.01 or ***P<0.001 comparing sh-ArfGAP1 with sh-control for G2019S LRRK2, or by comparing control with G2019S LRRK2, by one-way ANOVA with Newman-Keuls post-hoc analysis. *ns*, non-significant.
(TIF)

Figure S8 Increased neurite length induced by silencing of endogenous LRRK2 expression is not dependent on endogenous ArfGAP1. (A) Primary cortical neurons were co-infected with equal titers of lentiviral vectors expressing shRNAs (LV-sh-control, LV-sh-LRRK2- or LV-sh-ArfGAP1 #2) at DIV 2, subsequently transfected with a GFP construct at DIV 3 to morphologically label individual neurons, and fixed at DIV 6. Fluorescent microscopic images reveal the labeling of cortical neurons with GFP. GFP images were pseudo-colored with ICA for neurite length measurements. Neuronal soma (*arrows*) and axonal processes (*arrowheads*) are indicated. Scale bars: 400 µm. (B) Analysis of GFP-positive axonal processes reveals a robust increase of axonal length induced by silencing of LRRK2 expression alone compared to a non-silencing control shRNA. Co-silencing of ArfGAP1 fails to influence the LRRK2 silencing-induced increase in axonal length compared to a control shRNA. Bars represent mean (± SEM) length of axons expressed as a percent of GFP alone (LV-sh-control) from 50 GFP-positive neurons from two independent cultures. ***P<0.001 by one-way ANOVA with Newman-Keuls post-hoc analysis. *ns*, non-significant.
(TIF)
