# Supplementary figures and images for: GTPase Activity and Neuronal Toxicity of Parkinson's Disease–Associated LRRK2 Is Regulated by ArfGAP1
Source: PLoS Genet. 2012 Feb 9;8(2):e1002526. doi: 10.1371/journal.pgen.1002526 (PMC3280333; doi:10.1371/journal.pgen.1002526)

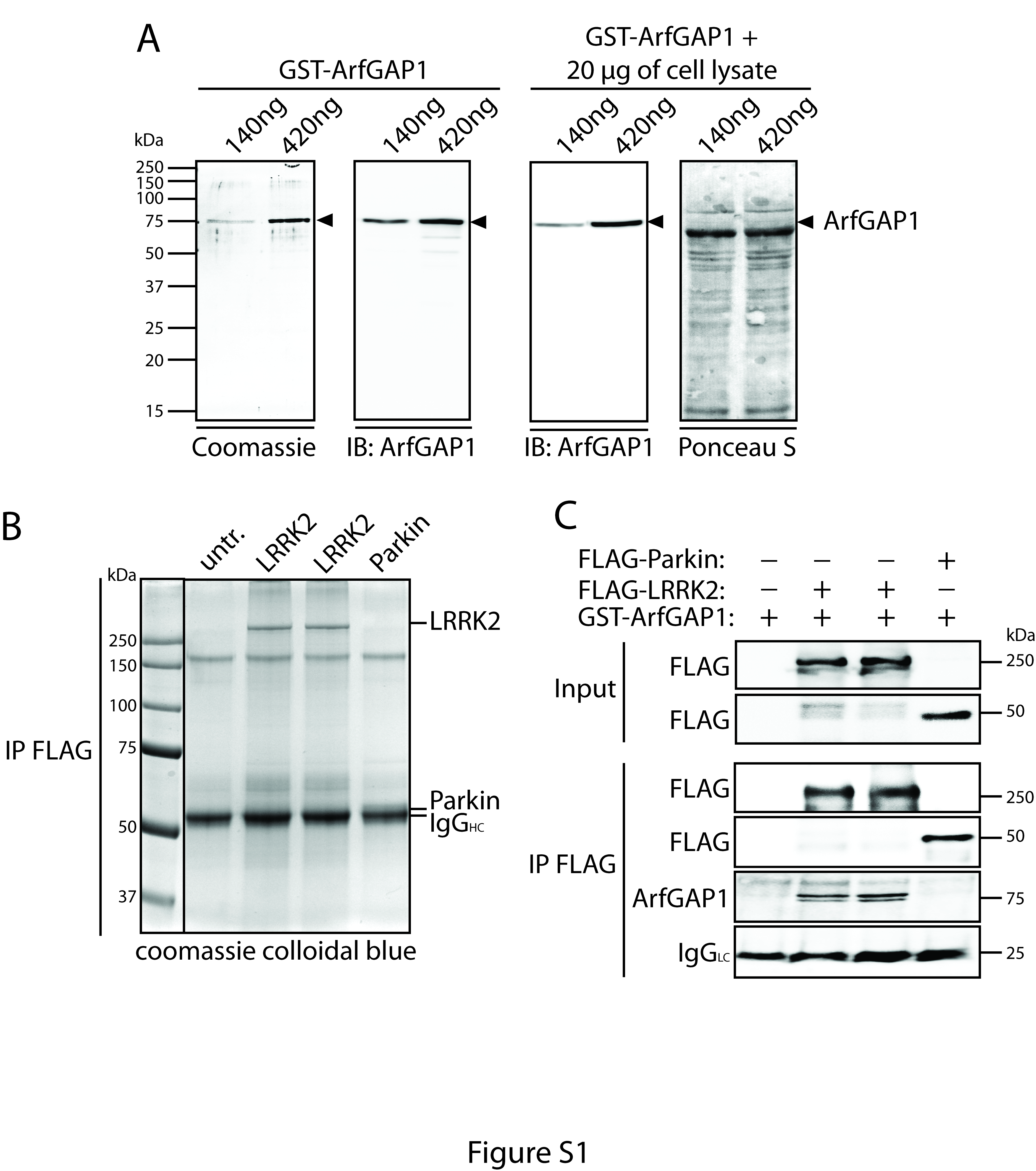

Supplement: Figure S1 — Direct interaction of LRRK2 with ArfGAP1. (A) SDS-PAGE analysis of recombinant full-length GST-tagged human ArfGAP1 (140 or 420 ng protein) by staining with coomassie colloidal blue or by Western blot analysis with rabbit anti-ArfGAP1 antibody (Proteintech Group Inc.). GST-ArfGAP1 was also added to HEK-293T cell lysates (20 µg protein) and detected by probing with anti-ArfGAP1 antibody. Equal loading of cell lysates is indicated by staining with Ponceau S. (B) SDS-PAGE analysis of recombinant FLAG-LRRK2 or FLAG-parkin immunoprecipitated (IP) from transfected HEK-293T cells with anti-FLAG antibody and stained with coomassie colloidal blue. IgG heavy chain (HC) is indicated confirming equal loading of IPs. FLAG IP from non-transfected (untr.) cell lysates was used to assess contaminating proteins (*). (C) In vitro interaction of recombinant GST-ArfGAP1 with immunopurified FLAG-LRRK2 but not with FLAG-parkin or mock FLAG IP. GST-ArfGAP1 interaction with each FLAG IP is detected with anti-ArfGAP1 antibody. IgG light chain (LC) is also indicated confirming equal loading of IPs. Molecular mass markers are indicated in kilodaltons. (TIF) [file pgen.1002526.s001.tif]

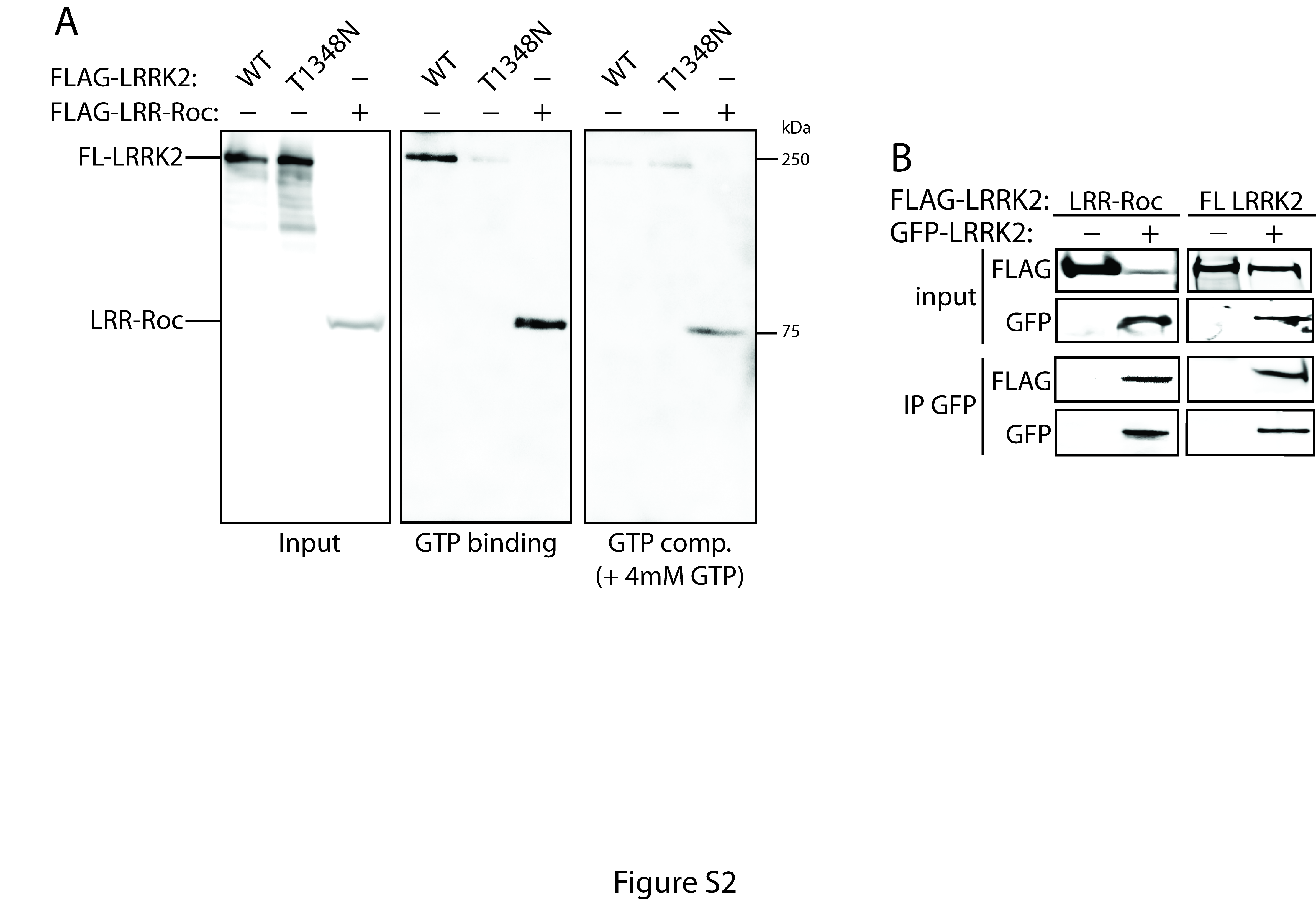

Supplement: Figure S2 — Confirmation of functional LRR-Roc protein fragment of LRRK2. (A) FLAG-tagged human LRR-Roc (fragment F3, residues 895–1503) and full-length WT LRRK2 bound to GTP following pull-down assays with GTP-sepharose from HEK-293T cells. Confirmation of the specificity of LRR-Roc or WT LRRK2 GTP binding is indicated by negligible binding of the GDP/GTP binding-deficient LRRK2 mutant, T1348N. Competition with an excess of free GTP (4 mM) reduces binding of LRR-Roc or WT LRRK2 to GTP-sepharose. (B) Co-immunoprecipitation of FLAG-tagged LRR-Roc or full-length WT LRRK2 with GFP-tagged full-length LRRK2 from HEK-293T cells following IP with anti-GFP antibody. Both LRR-Roc and WT LRRK2 are capable of forming dimers with GFP-LRRK2 suggesting appropriate folding of the LRR-Roc fragment. Molecular mass markers are indicated in kilodaltons. (TIF) [file pgen.1002526.s002.tif]

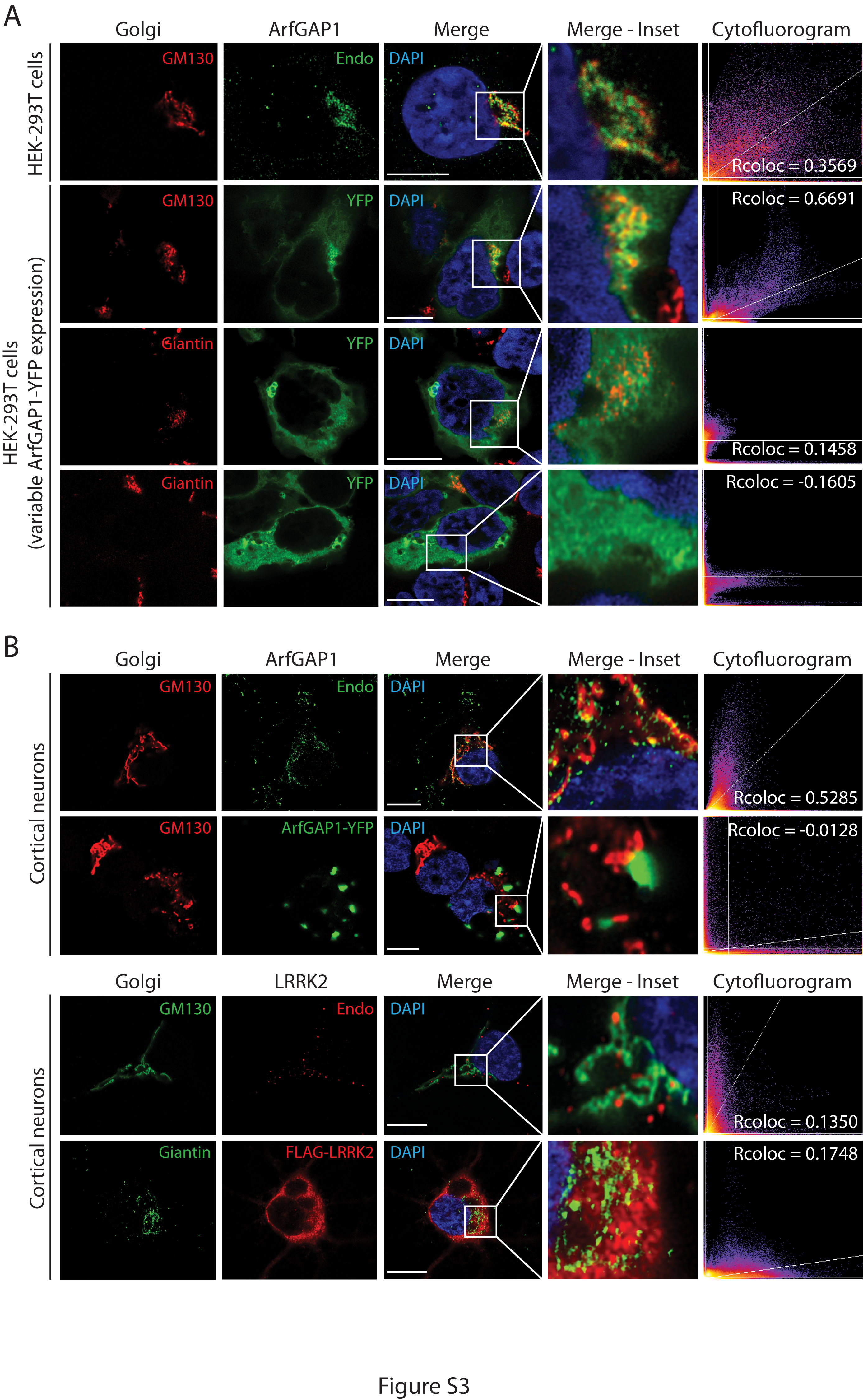

Supplement: Figure S3 — Co-localization of ArfGAP1 or LRRK2 with the Golgi complex in mammalian cells and neurons. (A) Confocal fluorescence microscopy reveals the co-localization of endogenous ArfGAP1 with the Golgi membrane marker, GM130, in HEK-293T cells. Exogenous ArfGAP1-YFP also co-localizes with Golgi membrane markers (GM130 or Giantin) to varying degrees depending upon the level of overexpression. ArfGAP1-YFP can localize to the intact Golgi complex (upper panel), can induce the fragmentation of the Golgi complex with the appearance of ArfGAP1-YFP-positive Golgi-derived vesicles (middle panel), or can induce the complete removal of the Golgi complex with ArfGAP1-YFP-positive Golgi-derived vesicles remaining (lower panel). (B) Similar co-localization of endogenous ArfGAP1 or exogenous ArfGAP1-YFP with the Golgi membrane marker, GM130, in rat primary cortical neurons (upper panels). ArfGAP1-YFP overexpression induces Golgi fragmentation with the appearance of ArfGAP1-YFP-positive Golgi-derived vesicles that are devoid of Golgi markers. Endogenous LRRK2 (JH5514 antibody) or exogenous FLAG-tagged LRRK2 partially co-localizes with the Golgi membrane markers, GM130 and Giantin, respectively, in primary cortical neurons (lower panels). Cytofluorograms and co-localization coefficients (Rcoloc) reveal the extent of co-localization between LRRK2 or ArfGAP1 and Golgi marker fluorescence signals. Confocal images are taken from single z-plane at 0.1 µm thickness. Images are representative of at least three independent experiments. Scale bars: 10 µm. (TIF) [file pgen.1002526.s003.tif]

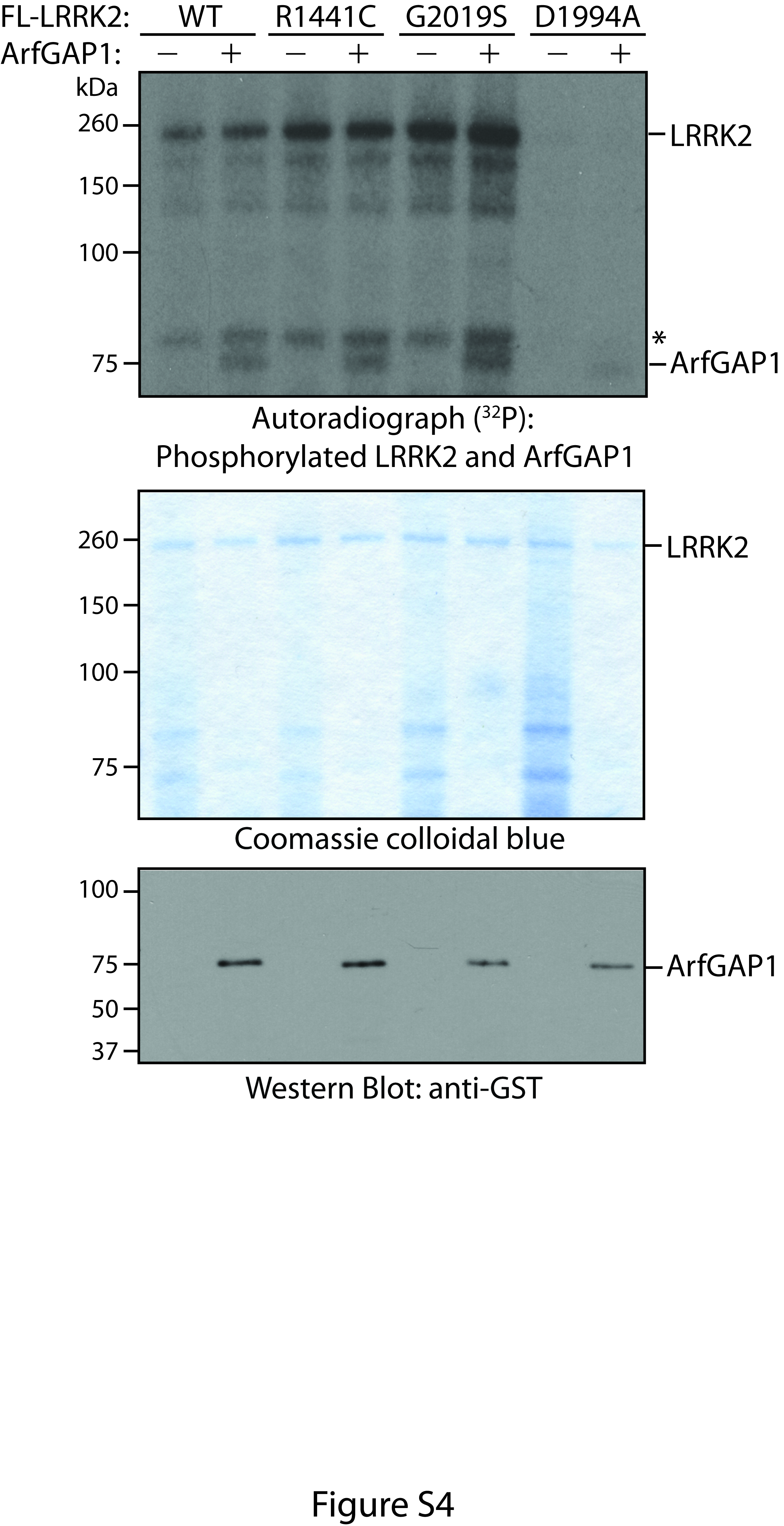

Supplement: Figure S4 — Full-length LRRK2 phosphorylates ArfGAP1. In vitro radioactive kinase assay with full length (FL) immunopurified myc-tagged human LRRK2 variants (WT, R1441C, G2019S and kinase-dead D1994A) and recombinant full-length GST-tagged human ArfGAP1. Autoradiographs (32P) reveal ArfGAP1 phosphorylation by WT, R1441C and G2019S LRRK2 but not D1994A LRRK2, with ArfGAP1 phosphorylation levels correlating with levels of LRRK2 autophosphorylation (upper panel). (*) indicates the LRRK2-dependent phosphorylation of an unknown LRRK2-interacting protein (>75 kDa) in these reactions. Coomassie colloidal blue-stained SDS-PAGE gels reveal equivalent loading and purity of immunoprecipitated full-length myc-LRRK2 variants (∼260 kDa) in each reaction (middle panel). Western blot analysis with anti-GST antibody indicates equivalent loading of GST-ArfGAP1 (∼75 kDa) in each reaction (lower panel). Molecular mass markers are indicated in kilodaltons. (TIF) [file pgen.1002526.s004.tif]

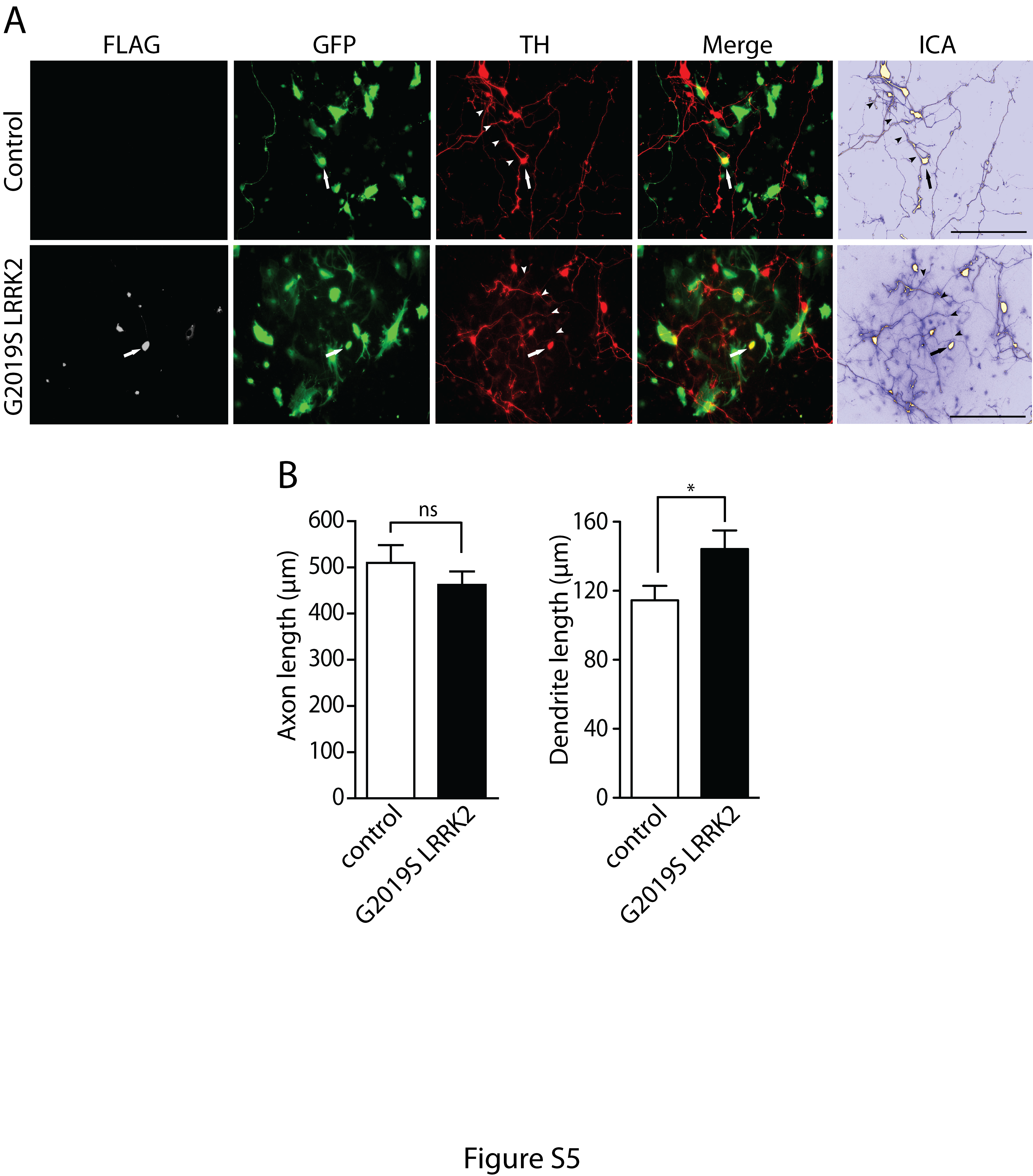

Supplement: Figure S5 — Effects of G2019S LRRK2 expression on neurite length of midbrain dopaminergic neurons. (A) Rat primary ventral midbrain cultures were transfected with FLAG-LRRK2 G2019S and GFP constructs at a 10∶1 molar ratio at DIV 3 and fixed at DIV 6. Fluorescent microscopic images indicate the co-labeling of FLAG-LRRK2, GFP and tyrosine hydroxylase (TH). GFP/TH merged images were rendered in ICA for neurite length measurements. Neuronal soma (arrows) and axonal processes (arrowheads) are indicated. Scale bars: 400 µm. (B) G2019S LRRK2 expression has negligible effects on the length of GFP/TH-positive dopaminergic axons but increases the length of dopaminergic dendrites, compared to GFP alone (control). Bars represent the mean (± SEM) length of axons or dendrites in µm from 30–40 GFP/TH-positive dopaminergic neurons from at least two independent experiments. *P<0.05 compared to control (GFP alone) by two-tailed unpaired Student's t-test. ns, non-significant. (TIF) [file pgen.1002526.s005.tif]

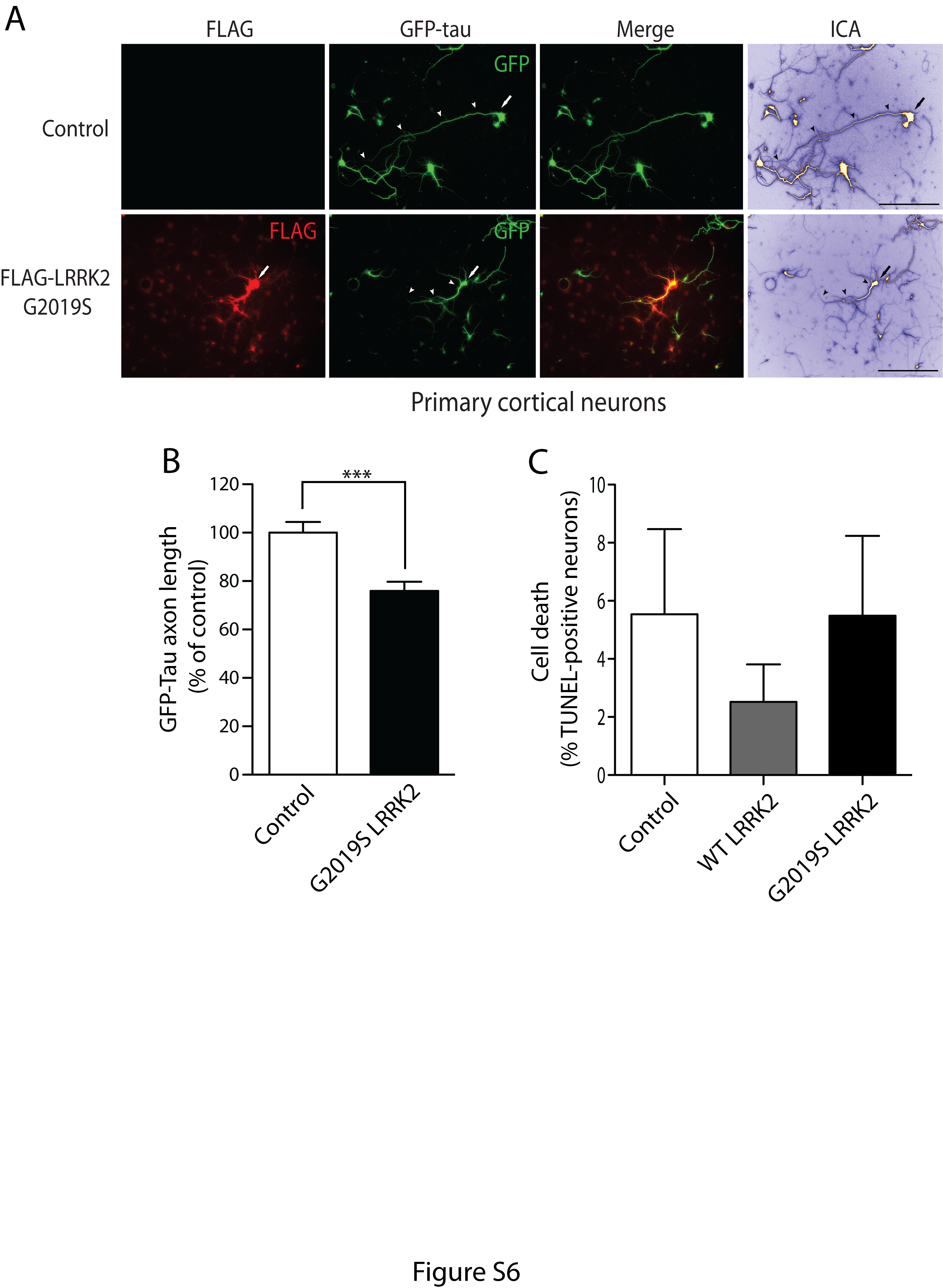

Supplement: Figure S6 — Effects of G2019S LRRK2 expression on axonal length and cell death in cortical neurons. (A) Rat primary cortical neurons were transfected at DIV 3 with FLAG-LRRK2 G2019S and GFP-tau constructs at a DNA molar ratio of 10∶1. Cultures were fixed at DIV 6. Fluorescent microscopic images reveal the co-labeling of cortical neurons with FLAG-LRRK2 and GFP-tau. GFP images were pseudo-colored with ICA to improve the contrast of axonal processes for length measurements. Neuronal soma (arrows) and axonal processes (arrowheads) are indicated. Scale bars: 400 µm. (B) Analysis of the length of GFP-tau-positive axonal processes reveals a significant shortening of axons due to G2019S LRRK2 expression, compared to GFP-tau alone (control). Bars represent axon length (mean ± SEM) expressed as a percent of control (GFP-tau alone) from 76–87 GFP-tau-positive neurons from at least two independent experiments/cultures. ***P<0.001 compared to control (GFP-tau alone) by two-tailed unpaired Student's t-test. (C) Effect of LRRK2 expression on apoptotic cell death of cortical neurons. Primary cortical neurons were transfected with FLAG-LRRK2 variants and GFP at a 10∶1 molar ratio at DIV 11 and fixed at DIV 14. Cultures were subjected to TUNEL staining and immunocytochemistry with anti-FLAG antibody. TUNEL-positive neurons were counted as a percent of total GFP-positive (control) or GFP/FLAG-positive (LRRK2) neurons and expressed as mean ± SEM (n = 3 experiments/cultures). (TIF) [file pgen.1002526.s006.tif]

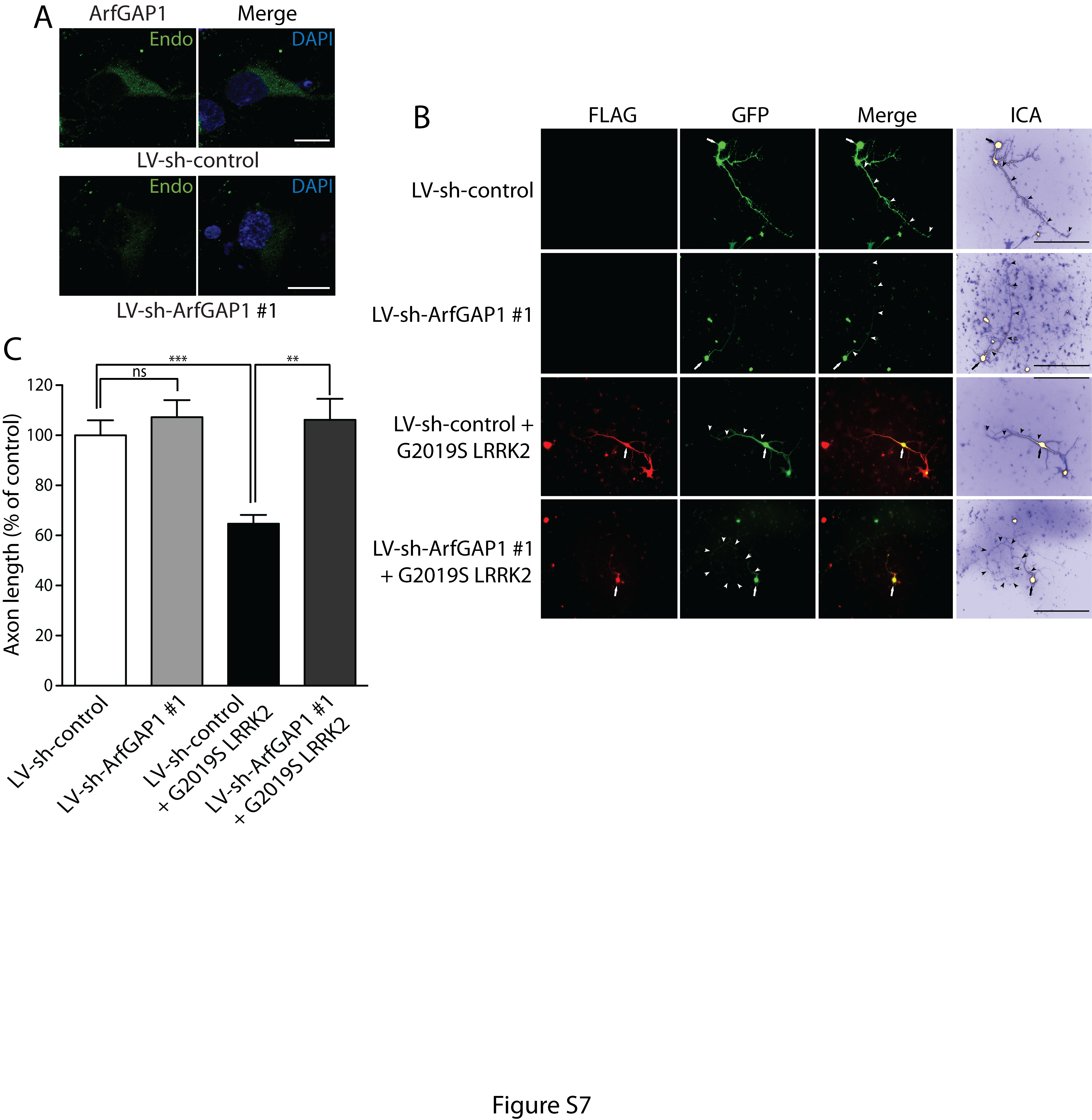

Supplement: Figure S7 — Silencing of ArfGAP1 expression with lentiviral sh-ArfGAP #1 rescues G2019S LRRK2-induced neurite shortening. (A) Silencing of endogenous ArfGAP1 expression in cortical neurons with a lentiviral ArfGAP1-specific shRNA (LV-sh-ArfGAP1 #1) compared to a non-silencing control shRNA (LV-sh-control) revealed by confocal fluorescence microscopy with a rabbit anti-ArfGAP1 antibody. (B) Primary cortical neurons were infected with lentiviral vectors expressing shRNAs (non-silencing control or ArfGAP1-specific) at DIV 2, subsequently transfected with FLAG-LRRK2 G2019S and GFP constructs at a 10∶1 molar ratio at DIV 3, and fixed at DIV 6. Fluorescent microscopic images reveal the co-labeling of cortical neurons with FLAG-LRRK2 and GFP. GFP images were pseudo-colored with ICA for neurite length measurements. Neuronal soma (arrows) and axonal processes (arrowheads) are indicated. Scale bars: 400 µm. (C) Analysis of GFP-positive axonal processes reveals a robust shortening of axons induced by G2019S LRRK2 expression compared to GFP alone (control/LV-sh-control). Knockdown of ArfGAP1 with lentiviral-shRNA vectors (LV-sh-ArfGAP1 #1) produces a complete rescue of G2019S LRRK2-induced axon shortening compared to control shRNA (LV-sh-control). Bars represent axon length (mean ± SEM) expressed as a percent of GFP alone (control/LV-sh-control) from >60 GFP-positive neurons from at least two independent experiments/cultures. **P<0.01 or ***P<0.001 comparing sh-ArfGAP1 with sh-control for G2019S LRRK2, or by comparing control with G2019S LRRK2, by one-way ANOVA with Newman-Keuls post-hoc analysis. ns, non-significant. (TIF) [file pgen.1002526.s007.tif]

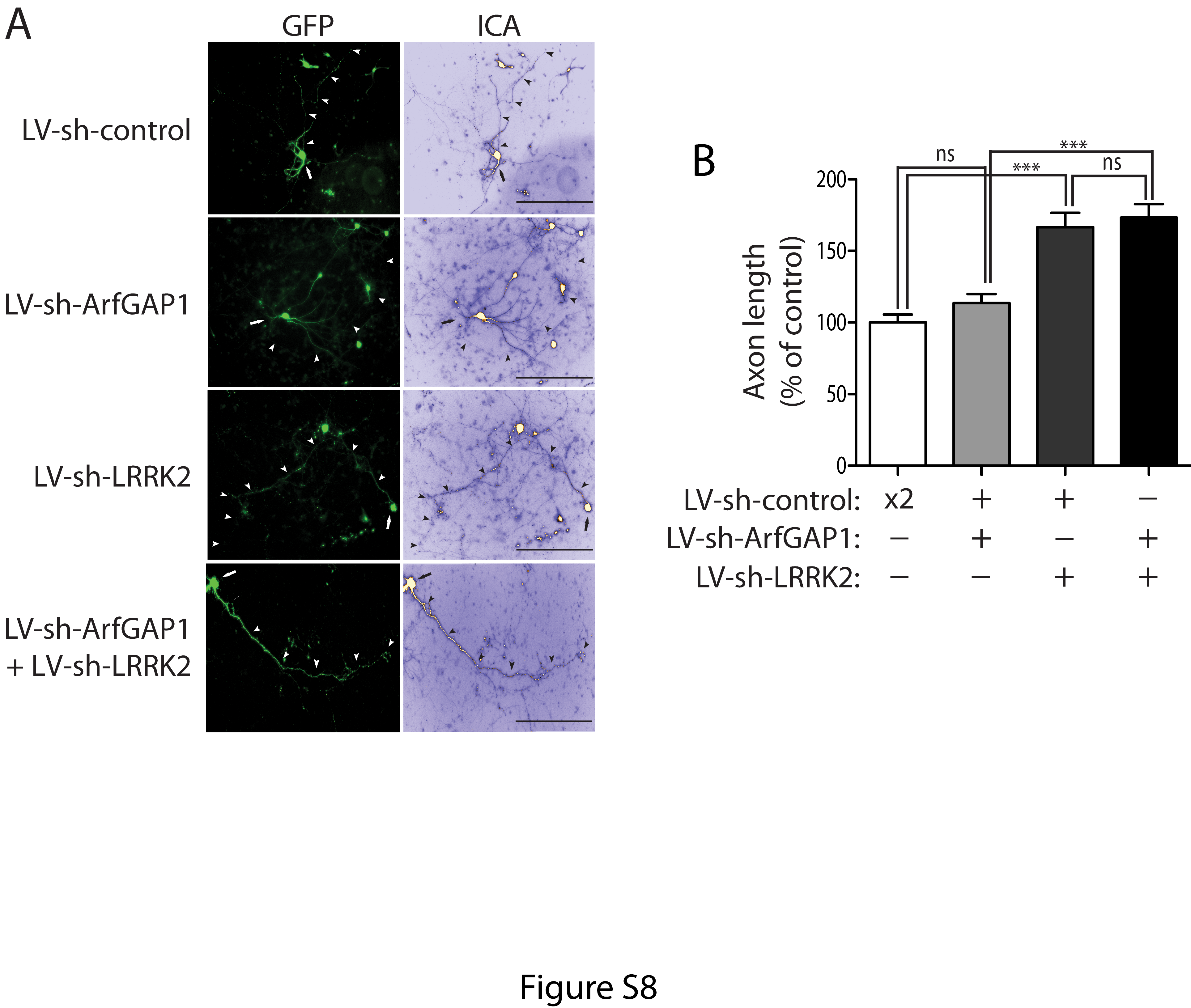

Supplement: Figure S8 — Increased neurite length induced by silencing of endogenous LRRK2 expression is not dependent on endogenous ArfGAP1. (A) Primary cortical neurons were co-infected with equal titers of lentiviral vectors expressing shRNAs (LV-sh-control, LV-sh-LRRK2- or LV-sh-ArfGAP1 #2) at DIV 2, subsequently transfected with a GFP construct at DIV 3 to morphologically label individual neurons, and fixed at DIV 6. Fluorescent microscopic images reveal the labeling of cortical neurons with GFP. GFP images were pseudo-colored with ICA for neurite length measurements. Neuronal soma (arrows) and axonal processes (arrowheads) are indicated. Scale bars: 400 µm. (B) Analysis of GFP-positive axonal processes reveals a robust increase of axonal length induced by silencing of LRRK2 expression alone compared to a non-silencing control shRNA. Co-silencing of ArfGAP1 fails to influence the LRRK2 silencing-induced increase in axonal length compared to a control shRNA. Bars represent mean (± SEM) length of axons expressed as a percent of GFP alone (LV-sh-control) from 50 GFP-positive neurons from two independent cultures. ***P<0.001 by one-way ANOVA with Newman-Keuls post-hoc analysis. ns, non-significant. (TIF) [file pgen.1002526.s008.tif]
